# A comprehensive study of hip dislocation: global health burden from 1990 to 2021 and its predictions to 2030

**DOI:** 10.3389/fpubh.2025.1594523

**Published:** 2025-09-09

**Authors:** Xu Zheng, Cheng Chen, Youguang Zhao, Jiantao Jiang, You Wang

**Affiliations:** ^1^Department of Bone and Joint Surgery, Department of Orthopedics, Renji Hospital, Shanghai Jiaotong University School of Medicine, Shanghai, China; ^2^Department of Orthopedics, Shanghai Tongji Hospital, School of Medicine, Tongji University, Shanghai, China; ^3^Department of Orthopedics, Shanghai Sixth People's Hospital Affiliated to Shanghai Jiao Tong University School of Medicine, Shanghai, China; ^4^Department of Orthopedics, Shaoxing Shangyu Traditional Chinese Medicine Hospital, Shaoxing, China

**Keywords:** epidemiology, Global Burden of Disease, incidence, hip dislocation, YLDs

## Abstract

**Objectives:**

Hip dislocation is a critical clinical emergency that demands immediate intervention to prevent complications such as avascular necrosis, nerve damage, and long-term joint dysfunction. As population demographics evolve—with aging populations and increasing urbanization—and injury patterns shift due to factors such as road traffic accidents, sports-related injuries, and occupational hazards, the need to update our understanding of the epidemiology of hip dislocation becomes ever more pressing. These changes highlight the importance of identifying high-risk groups and tailoring preventive and therapeutic strategies accordingly. This study leverages comprehensive data from the Global Burden of Disease (GBD) 2021 to analyze the latest trends, disease burden, and population-specific patterns of hip dislocation. This analysis aims to produce evidence-based insights that inform clinical practice, guide public health policies, and promote efficient resource allocation. The findings will also help predict future trends, enabling proactive measures to mitigate the impact of hip dislocation on global health.

**Methods:**

Data from the GBD 2021 was utilized to calculate the estimated annual percentage change in hip dislocation metrics. A comprehensive analysis of population patterns was conducted, focusing specifically on region, age and gender distributions. Key measures included crude and age-standardized incidence rates, crude and age-standardized Years Lived with Disability (YLDs) rates, and absolute case numbers and corresponding 95% uncertainty intervals (UIs). The estimated annual percentage change (EAPC) and corresponding 95% confidence intervals (CIs) were calculated, with positive EAPC values indicating an increasing trend and negative values indicating a decreasing trend. Socio-Demographic Index (SDI) was cross-analyzed with other study indicators to examine potential correlations. Bayesian Age-Period-Cohort models, implemented through the BAPC R package, were used to project changes in the disease burden of hip dislocation by 2030.

**Results:**

From 1990 to 2021, the incidence rate and YLDs rate of hip dislocation decreased, while the number of cases and YLDs number increased. The disease burden is relatively high among the youth (15–24) and the older population (50+). In SDI-stratified analyses, incidence gaps narrowed across all quintiles, yet low-SDI regions still bore a comparatively higher residual disability burden by 2020. Males have consistently faced a heavier burden than females. High-energy trauma, particularly falls, warrants special attention in the older population. The total number of hip dislocation cases will increase, while the corresponding rate declines. Men will still bear a substantial disease burden of hip dislocation by 2030.

**Conclusion:**

The disease burden of hip dislocation remains a significant public health challenge. Data-driven analysis is pivotal for guiding clinical practice, shaping public health policies, and ensuring optimal allocation of healthcare resources. Strategic efforts are needed to address the persisting burden, particularly among high-risk demographic groups.

## 1 Introduction

Traumatic hip dislocation, defined as the displacement of the femoral head from the acetabulum due to external forces, represents a critical orthopedic emergency demanding prompt intervention to prevent serious complications. Falls and traffic accidents are recognized as the leading cause of this injury, particularly in both younger adults and the older population (1).

Posterior dislocations are the most common, accounting for 80–90% of cases (2–4). Regardless of the specific type, traumatic hip dislocations carry significant risks of complications such as avascular necrosis, post-traumatic arthritis, sciatic nerve injury, and joint instability (5–8). Though closed reduction is the standard treatment, complex cases involving fractures or delayed presentation may require surgical intervention. Long-term complications, including avascular necrosis and post-traumatic arthritis, are substantial challenges, with outcomes closely tied to the severity of injury and time to reduction (9). Early and effective management is essential to mitigate these outcomes and restore joint function.

Although more frequent in younger, working individuals, a secondary demographic peak is observed in older adults, primarily due to falls (1, 10, 11). With an increasing older population, understanding injury patterns and optimizing care for this group have become more urgent (12). Due to demographic changes and changes in injury patterns especially brought about by social development including capita income, educational attainment, and total fertility rate, it is necessary to understand the latest epidemiology of hip dislocation (13).

In this study, we utilized Global Burden of Disease (GBD) data and Bayesian Age-Period-Cohort (BAPC) models to comprehensively analyze the trends and future predictions of hip dislocation from 1990 to 2021, with projections to 2030. The GBD data was chosen due to its extensive coverage of epidemiological metrics across different regions, populations, and time periods, offering a robust and standardized dataset for assessing the global health burden of hip dislocation (14). Additionally, the BAPC models were selected for their ability to account for complex temporal patterns by disentangling the effects of age, period, and cohort. This approach is particularly valuable for understanding the underlying drivers of hip dislocation trends, such as demographic changes and healthcare advancements, and for making reliable future predictions (15, 16).

Studying the incidence and disability rates of traumatic hip dislocation holds significant importance as it sheds light on a substantial yet often underestimated health burden. This research presents an extensive epidemiological study of hip dislocations from 1990 to 2021, aiming to elucidate demographic and geographic variations across this timeframe, ultimately advancing global public health strategies and informing more targeted clinical resource allocation.

## 2 Methods

The Global Burden of Disease (GBD) study, initiated by the Institute for Health Metrics and Evaluation (IHME), is an extensive global health initiative that systematically quantifies the health impacts of various diseases, injuries, and risk factors across the world. Serving as a critical resource for policymakers, researchers, and global health stakeholders, the GBD provides a comprehensive framework for understanding global health patterns and supports evidence-based decision-making. The most recent iteration, GBD 2021, incorporates updated data and methodological advancements, reflecting the evolving nature of global health trends (GBD 2021 Data Resources). This version offers valuable insights to inform public health policies, guide research priorities, and support international development efforts.

The GBD 2021 database uses multiple data sources to estimate years lived with disability, years of life lost, disability-adjusted life years and healthy life expectancy for 371 diseases and injuries (14). The data from GBD 2021 are publicly accessible and can be retrieved from the official website.^1^ For the present analysis, data on incidence and YLDs, as well as their corresponding 95% UIs related to hip dislocation from 1990 to 2021 were extracted from the GBD 2021 database. Hip fractures were excluded from hip dislocations in this study. Socio-Demographic Index (SDI) is a standardized composite index developed by GBD researchers that quantifies socioeconomic development and population demographics across nations by integrating three key indicators: per capita income, mean educational attainment, and total fertility rate (14). SDI values range between 0 and 1, with 0 denoting minimal health development and 1 representing optimal health development in theoretical terms. SDI was integrated with additional study metrics for comprehensive analysis.

### 2.1 Statistical analysis

A systematic analysis of the global burden of hip dislocation was performed utilizing the GBD 2021 dataset. Incidence, YLDs and population data were obtained from the GBD Results Tool, available at https://vizhub.healthdata.org/gbd-results. The Estimated Annual Percentage Change (EAPC) serves as a quantitative measure of temporal trends in age-standardized rates (ASR). To calculate this metric, the natural logarithm of ASR was modeled using a linear regression framework: ln (ASR) = *α* + *β*x + *ε*, where α represents the intercept, β denotes the annual slope coefficient representing the change in ln-transformed rates per calendar year (x), and ε signifies the error term. The EAPC was derived using the formula 100 × (exp (β)-1), reflecting the annual percentage change in ASR (17, 18). The corresponding 95% confidence interval was calculated through propagation of the standard error estimates obtained from the regression parameters. BAPC model decomposes the observed rates (𝑦𝑎,𝑝) into additive components for age (𝛼𝑎), period (𝛽𝑝), and cohort (𝛾𝑐) effects, subject to smoothness constraints to address identifiability issues (19). Second-order random walk (RW2) priors were used for these components, ensuring smooth transitions across age, period, and cohort groups. Posterior inference was performed using Markov Chain Monte Carlo (MCMC) methods, providing credible intervals for the estimated effects. Furthermore, detailed demographic analyses were conducted to examine the patterns of hip dislocation by age and gender.

All data analysis and visualization were carried out using R software (version 4.2.2). Bayesian Age-Period-Cohort models, implemented through the BAPC R package, were used to project changes in the disease burden of hip dislocation by 2030.

## 3 Results

### 3.1 The burden of hip dislocation at the global level

**Figure 1:**
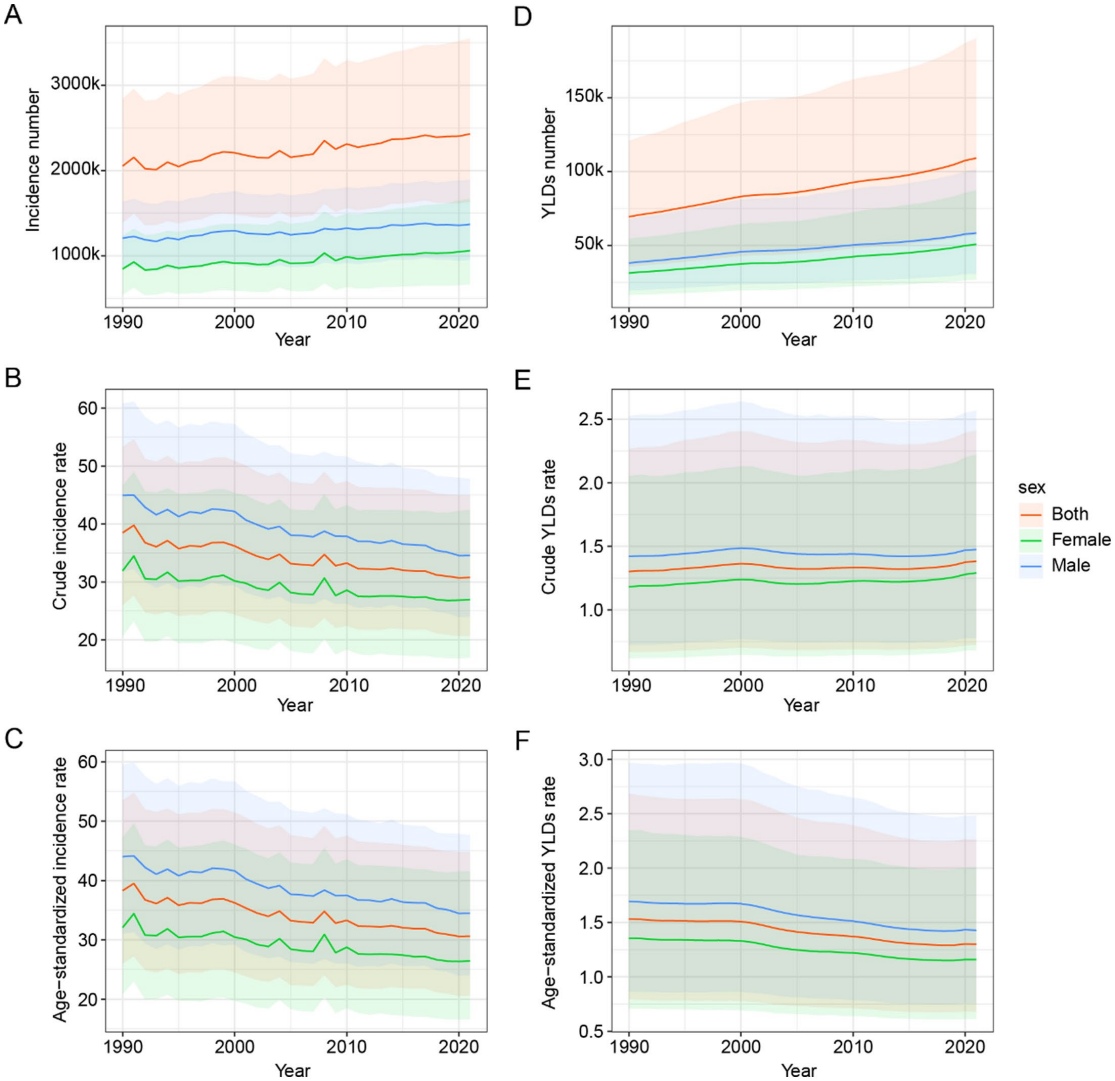
Global epidemiological trends of hip dislocation across gender from 1990 to 2021. **(A)** Incidence number. **(B)** Crude incidence rate. **(C)** Age-standardized incidence rate. **(D)** YLDs number. **(E)** Crude YLDs rate. **(F)** Age-standardized YLDs rate. YLDs, years lived with disability.

From 1990 to 2021, a global upward trend was observed in the incidence number of hip dislocations, while the crude incidence rate experienced a decline. The age-standardized incidence rate (ASIR) also demonstrated a decline. The number of YLDs (Years Lived with Disability), along with the crude YLDs rate and the age-standardized YLDs rate, exhibited trends that closely mirrored those of the incidence figures, the crude incidence rate, and the age-standardized incidence rate, respectively. Notably, males consistently exhibited a heavier burden compared to females, as illustrated in Figure 1.

From 1990 to 2021, there was a noticeable increase in the global incidence number of hip dislocation. The global incidence number of hip dislocation escalated from 2,052,924 (95% UI: 1,388,083 to 2,841,632) in 1990 to 2,429,935 (95% UI: 1,634,456 to 3,549,251) in 2021. The crude incidence rate also showed a decrease from 38.49 (95% UI: 26.03 to 53.28) in 1990 to 30.79 (95% UI: 20.71 to 44.98) in 2021. Similarly, the ASIR exhibited a downward trend, decreasing from 38.29 (95% UI: 26.02 to 53.53) in 1990 to 30.63 (95% UI: 20.65 to 44.80) in 2021.

In terms of YLDs, there was a significant increase in the global YLDs number for hip dislocation from 69,430 (95% UI: 35,716 to 120,858) in 1990 to 109,145 (95% UI: 57,180 to 190,161) in 2021. The crude YLDs rate slightly increased from 1.30 (95% UI: 0.67 to 2.27) in 1990 to 1.38 (95% UI: 0.72 to 2.41) in 2021. Conversely, the age-standardized YLDs rate showed a decline from 1.53 (95% UI: 0.80 to 2.68) in 1990 to 1.30 (95% UI: 0.68 to 2.26) in 2021.

### 3.2 The burden of hip dislocation at the national and territory level

The countries and territories with the highest EAPC in the ASIR of hip dislocation were the Syrian Arab Republic (7.84, 95% CI: 5.1 to 10.66), Yemen (3.86, 95% CI: 2.61 to 5.12), and Libya (2.73, 95% CI: 1.3 to 4.18). In 2021, the countries and territories with the highest ASIR were Afghanistan (157.41, 95% UI: 78.59 to 322.83), Yemen (93.92, 95% UI: 53.04 to 182.95), and Slovenia (84.43, 95% UI: 51.6 to 131.97).

**Figure 2:**
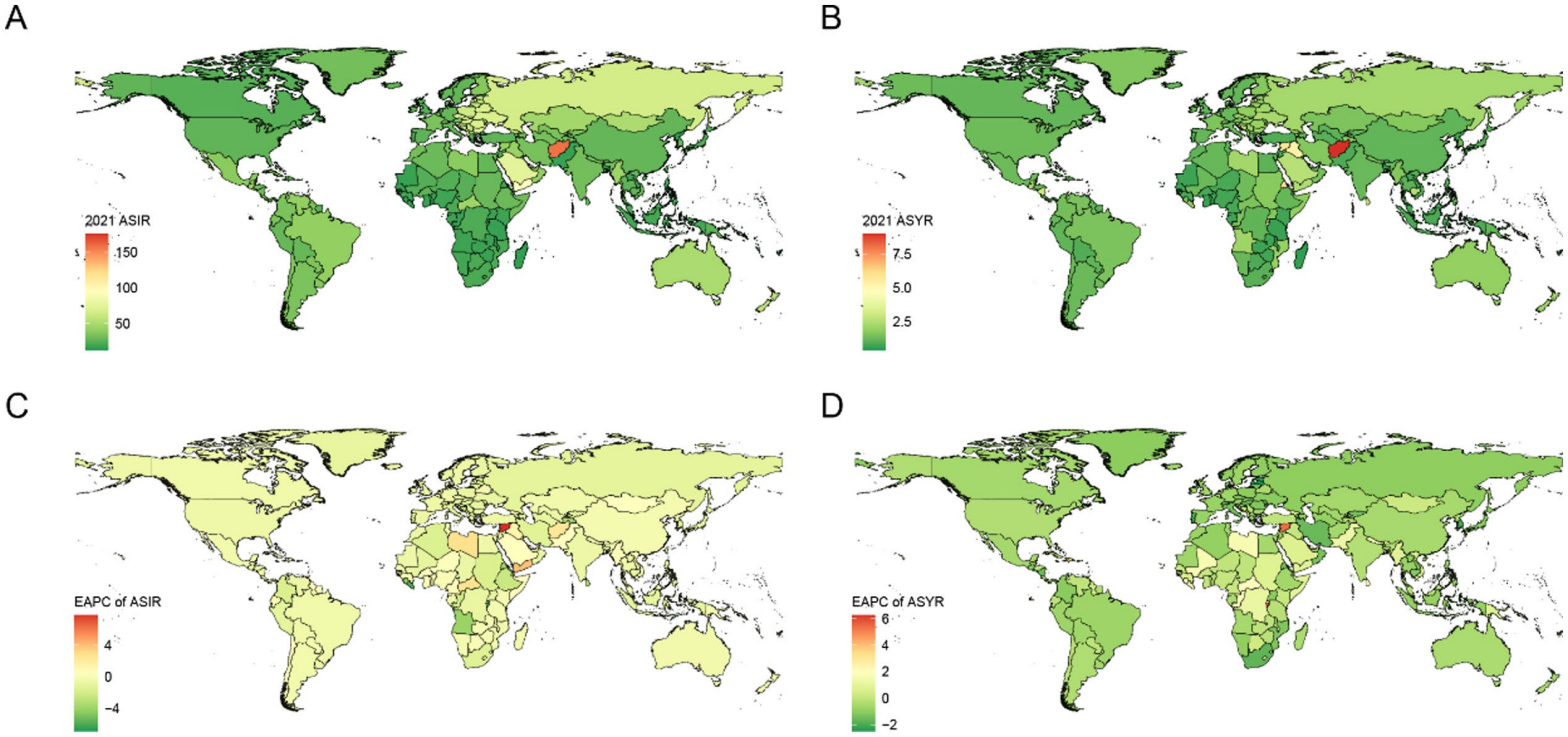
ASIR **(A)** and ASYR **(B)** maps of hip dislocation by countries and territories in 2021. EPAC maps of ASIR **(C)** and ASYR **(D)** of hip dislocation by countries and territories from 1990 to 2021. YLDs, years lived with disability; ASIR, age-standardized incidence rate; ASYR, age-standardized YLDs rate; EAPC, estimated annual percentage change.

The countries and territories with the highest EAPC in the age-standardized YLDs rate were Burundi (6.27, 95% CI: 4.42 to 8.15), Syrian Arab Republic (5.35, 95% CI: 3.84 to 6.88), and Haiti (4.57, 95% CI: 3.45 to 5.71). In 2021, the countries and territories with the highest age-standardized YLDs rate were Afghanistan (9.03, 95% UI: 2.61 to 24.67), Syrian Arab Republic (5.85, 95% UI: 2.17 to 12.88), and Eritrea (5.84, 95% UI: 1.87 to 14.76). For detailed data, see Supplementary Tables S1, S2. Visualization can be seen in Figure 2.

### 3.3 The burden of hip dislocation at the socioeconomic level

**Figure 3:**
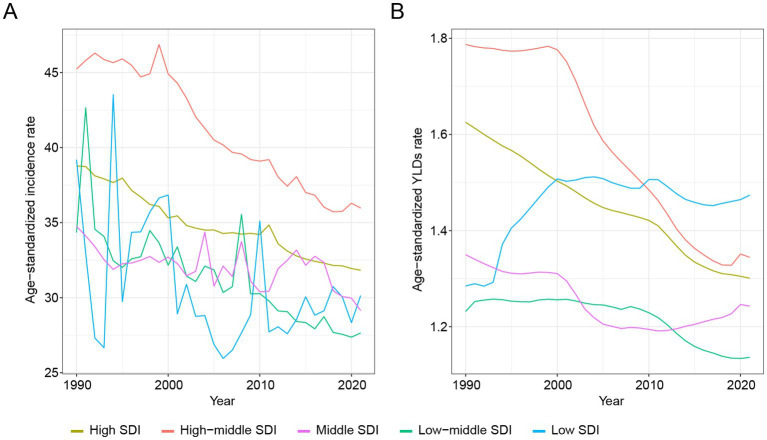
SDI-quintile-stratified temporal trends of hip dislocation in ASIR **(A)** and ASYR **(B)** from 1990 to 2021. SDI, Socio-Demographic Index; ASIR, age-standardized incidence rate; ASYR, age-standardized YLDs rate.

In a stratified analysis by SDI quintile, a pronounced socioeconomic gradient in hip dislocation incidence was observed in Figure 3. In 1990, the highest age-standardized incidence rates were recorded in high-SDI regions and the lowest in low-SDI regions, with high-middle, middle and low-middle regions occupying intermediate positions. Although all quintiles exhibited reduced age-standardized incidence rates over the study period, the magnitude of decline was greatest in high-middle and low-middle SDI regions, intermediate in high and low SDI regions, and smallest in the middle SDI regions. Middle-, low-middle-, and low-SDI regions showed marked fluctuations or rebounds, whereas high- and high-middle-SDI regions exhibited a steadier decline. As a result, the gap in incidence between the highest and lowest SDI groups narrowed by the end of the period.

An analogous pattern emerged for age-standardized YLDs rates. Regions in the high and high-middle SDI quintiles—initially bearing the greatest disability burden—achieved continuous decreases, while middle and low-middle SDI regions experienced more gradual reductions. In contrast, low-SDI regions displayed an initial increase in YLDs rates before plateauing and then modestly declining, culminating in a relatively higher residual burden by 2020.

### 3.4 Age and gender patterns of hip dislocation

**Figure 4:**
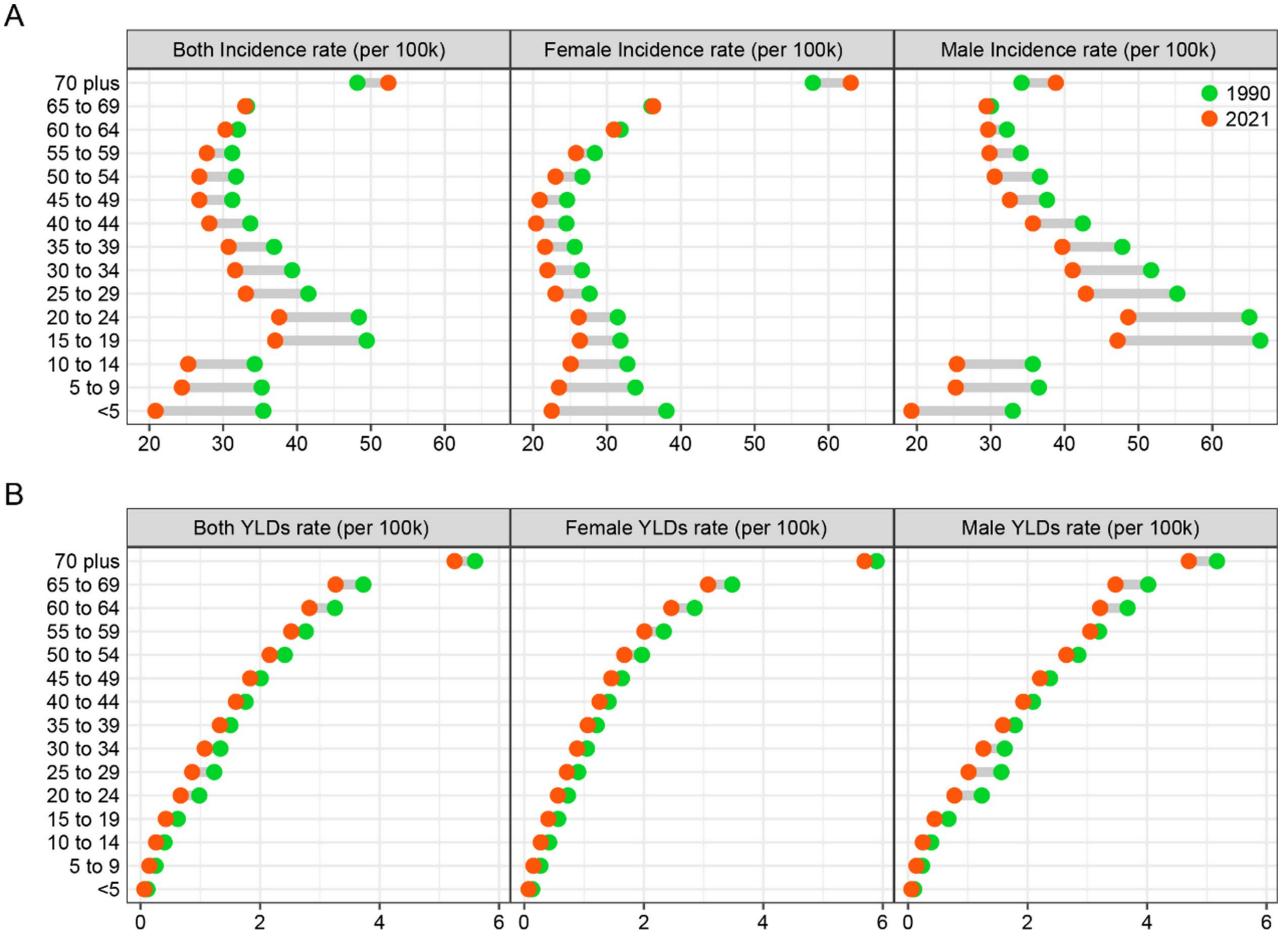
The change of global incidence **(A)** and YLDs **(B)** rates of hip dislocation across age and gender from 1990 to 2021. YLDs, years lived with disability.

Figure 4 illustrates the age and gender patterns of the disease burden associated with hip dislocations. For the entire population, the incidence rate of hip dislocations peaks between the ages of 15–24 and then declines with increasing age, only to rise again after the age of 50. For females, there is a difference in the incidence rate patterns between 1990 and 2021. In 1990, the incidence rate gradually decreased from birth, beginning to rise again after the age of 45. In contrast, in 2021, the peak incidence rate was reached between the ages of 15–24, after which it declined with age, only to rise again after the age of 45. The epidemiological patterns for males in 1990 and 2021 are relatively consistent, with the highest peak occurring between the ages of 15–24, followed by a year-by-year decline. A slight difference is observed in 2021, where the incidence rate among patients aged 50 to 69 decreased very gradually. The incidence rate in 2021 was consistently lower than in 1990, except for patients over the age of 70, where there was a reversal, with the incidence rate in 2021 being higher than in 1990 for both the overall population and the two distinct gender groups.

In both the overall and the two distinct gender groups, YLDs rate showed a gradual increase with advancing age. We focus on the differences in YLDs rate between 1990 and 2021. Similar to the overall group, the female group exhibits the largest difference in YLDs rate in the 65–69 age group. However, for males, the greatest improvement in YLDs rate is observed in the 25–29 age group and the 65–69 age group. In the age group above 70, males show a larger improvement in YLDs rate, while females show a smaller improvement.

### 3.5 The leading causes of global incidence rates of hip dislocation

**Figure 5:**
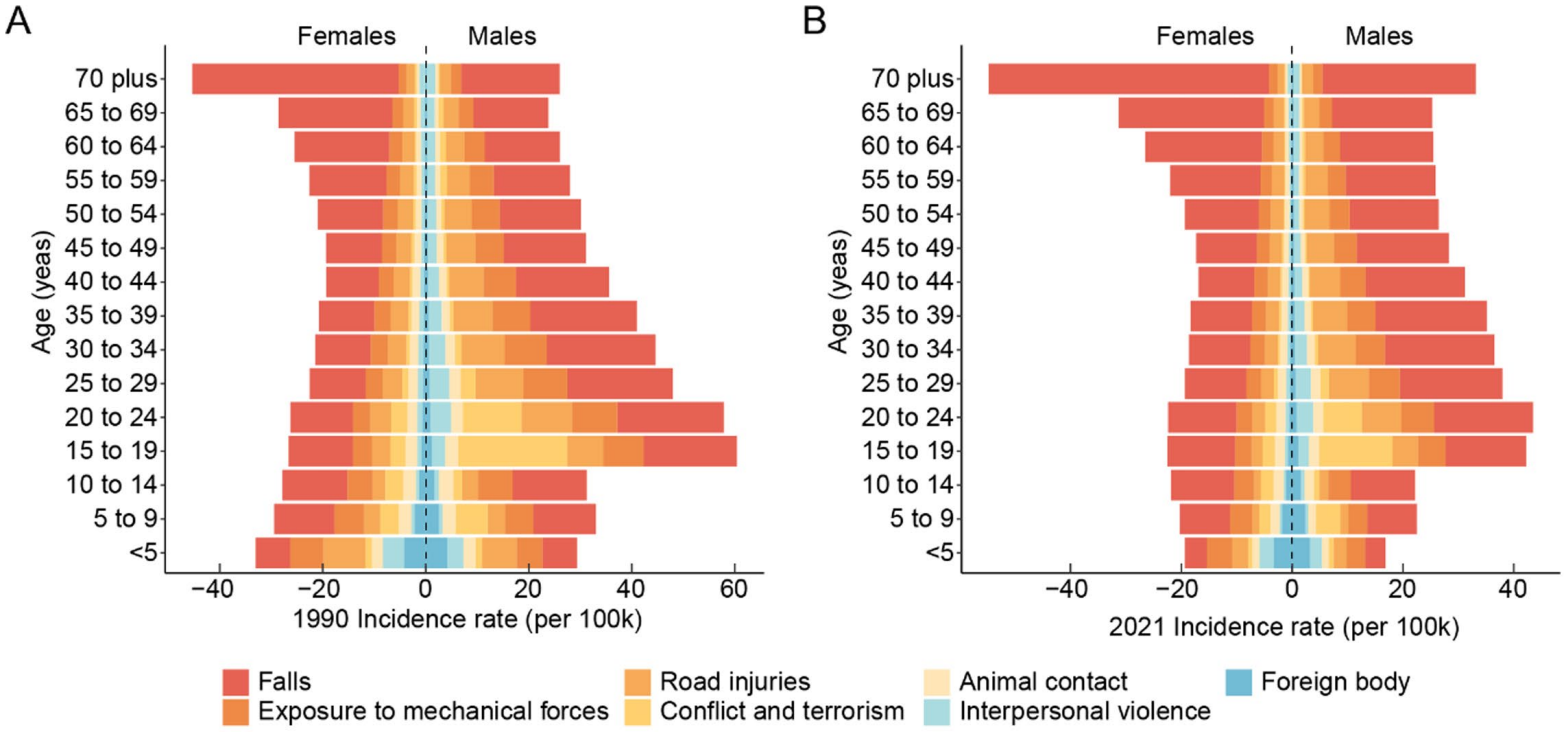
The leading causes of global incidence rates of hip dislocation across age and gender from 1990 **(A)** to 2021 **(B)**.

Figure 5 elucidates the causative factors contributing to hip dislocations across various genders and age groups between the years 1990 and 2021. Males consistently exhibit higher incidence rates than females across most age groups, particularly in younger and middle-aged populations. In 2021, the leading causes of hip dislocations were road injuries, exposure to mechanical forces, falls, and conflict and terrorism. The age-standardized incidence rates for hip dislocations for both sexes were as follows: road injuries, 3.21 (95% UI: 1.63 to 6.08); exposure to mechanical forces, 3.49 (95% UI: 1.26 to 7.53); falls, 14.77 (95% UI: 7.14 to 27.67); and conflict and terrorism, 1.75 (95% UI: 0.70 to 4.01). The age-standardized YLDs rates were: exposure to mechanical forces, 0.06 (95% UI: 0.03 to 0.11); road injuries, 0.19 (95% UI: 0.10 to 0.33); falls, 0.58 (95% UI: 0.30 to 1.00); and conflict and terrorism, 0.15 (95% UI: 0.05 to 0.39). Notably, compared with young adults (20–39 years), older adults (65 + years), especially females, experience higher incidence rates, primarily driven by falls.

Comparing the data from 1990 and 2021, there is a general reduction in the overall incidence rates across most age groups and causes, indicating improvements in safety and preventive strategies over the decades. Road injuries, which were a significant cause of hip dislocation among younger and middle-aged males in 1990, show a decline in 2021. Similarly, hip dislocations related to interpersonal violence and conflict have also decreased slightly, though they remain substantial contributors among young males. In contrast, falls among older adults, particularly females aged 65 and above, have increased in prominence in 2021, highlighting an ongoing challenge in mitigating fall-related injuries in aging populations.

### 3.6 Prediction of the burden of hip dislocation disease in 2030

**Figure 6:**
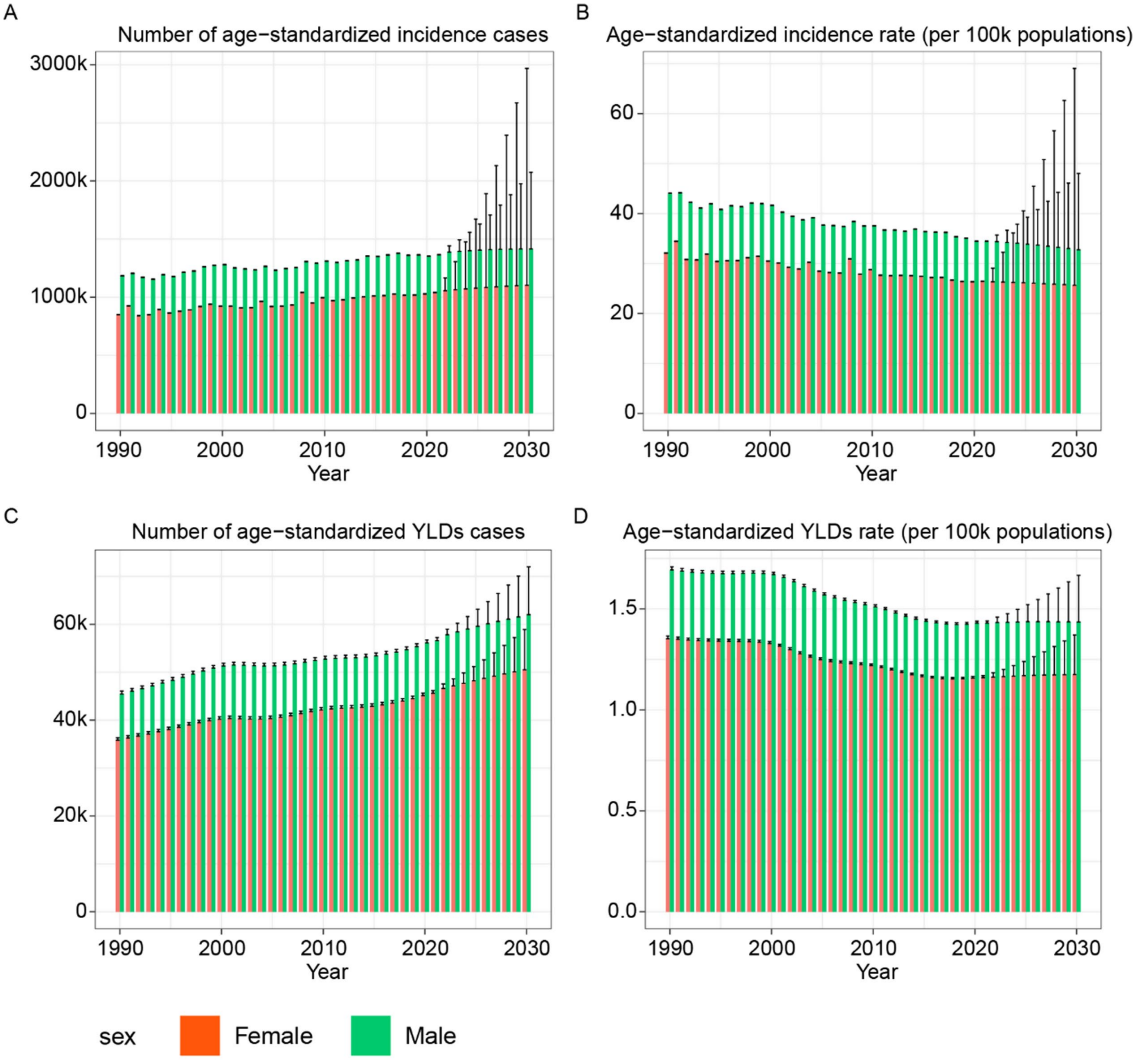
Prediction to 2030 of hip dislocation in age-standardized rate and number of incidence and YLDs. **(A)** Number of age-standardized incidence cases. **(B)** Age-standardized incidence rate. **(C)** Number of age-standardized YLDs cases. **(D)** Age-standardized YLDs rate. YLDs, years lived with disability.

The projected burden of hip dislocation by 2030 highlights a complex pattern of declining age-standardized rates but increasing total numbers of age-standardized incidence and YLDs in Figure 6. Although males and females followed similar temporal trends, values for all four metrics were consistently higher in males. Projections suggest that the gender gap in age-standardized rates will narrow by 2030. Over the previous decades, both males and females have shown consistent reductions in ASIR and age-standardized YLDs rates, and this trend is expected to continue. By 2030, ASIR for hip dislocation is predicted to decline to 32.76 for males and 25.65 for females. Similarly, the age-standardized YLDs rates are forecasted to decrease to 1.44 for males and 1.18 for females. In contrast, the total number of age-standardized incidence cases and YLDs is projected to rise significantly by 2030. Men are expected to experience an age-standardized incidence number of 1,414,993 and an age-standardized YLDs number of 61,999, compared to women, who are projected to have an age-standardized incidence number of 1,102,912 and an age-standardized YLDs number of 50,518.

## 4 Discussion

Studying the epidemiology of traumatic hip dislocation is crucial not only for quantifying its burden but also for driving impactful change across the spectrum of care. This research delves into the real-world implications for individuals and society, encompassing factors such as lost productivity, healthcare expenditures, and diminished quality of life. Such data plays a critical role in guiding resource allocation within healthcare systems, driving targeted prevention strategies by identifying risk factors, and assessing treatment efficacy. The innovation in this field lies in a shift towards more comprehensive outcome measures that consider disability, functional outcomes, and patient-reported data. This involves harnessing big data and technology for larger, more precise studies, focusing on specific populations like the older population or athletes, and employing advanced modeling and simulation techniques to predict future trends and enhance patient care.

Accurate descriptions of the incidence and disability rates of traumatic hip dislocation are limited in existing research articles. Comprehensive data on hip dislocation incidence is limited due to variations in data collection methodologies across studies, coupled with the relative rarity of these injuries, and the influence of confounding factors like age and activity levels make accurate estimations challenging (6, 7, 20). This study, categorized by age, gender, and regional development levels, analyzes the epidemiological changes in hip dislocation from 1990 to 2021. It provides assistance in evaluating past experiences and formulating further policies. Another difficulty lies in the fact that disability is often assessed indirectly through measures of complications (like Posttraumatic Arthritis and Avascular Necrosis) or functional scores, rather than a single “disability rate” statistic. Despite increasing focus on prognostic factors influencing the return to daily activities in patients with hip dislocation, reliable quantitative studies remain scarce—likely due to small sample sizes and challenges in maintaining prolonged, detailed follow-up (21, 22). Studies tend to focus on these specific outcomes rather than an overarching disability percentage.

This study utilized computational methods to analyze the epidemiological characteristics of traumatic hip dislocations, integrating insights from relevant literature and existing datasets. Computational modeling reveals a nuanced picture of global trends, where the incidence of hip dislocations shows a decline due to advancements in medical security systems and road safety measures. However, contrasting findings in some studies highlight an increase in the overall number of reported cases, particularly in developed countries (1). The observed contradiction between the decreasing crude incidence rate of hip dislocation and the increasing total number of cases can be explained by demographic changes, as exemplified by trends in China. Despite a declining crude incidence rate, China’s significant population growth has led to a larger total population, increasing the absolute number of cases even with a lower rate (23). Additionally, the country’s rapid population aging (especially in cities) has resulted in a growing proportion of older adults, who are at higher risk for hip dislocation (23). These aging population combined with rising morbidity imposes a substantial burden on health and social care systems (24). This demographic shift contributes to an overall rise in cases, as the older population expands, even if age-specific incidence rates are stable or decreasing as a result of health propaganda and education. These dual trends are shaped by population-wide improvements in risk reduction and diverse demographic factors influencing the burden of disease. For instance, in developed countries, urban population aging and the growing focus on traffic safety and diagnostic precision have contributed to both risk mitigation and improved detection (25–27). Advancements in diagnostic scoring systems have also improved the identification of hip dislocations, which may partially account for the observed increases in reported cases. Additionally, the rise in sports-related traumatic hip dislocations linked to activities such as soccer, skiing, and basketball underscores the importance of targeted preventative strategies for specific populations (28).

In developing countries, the analysis result highlights a different set of factors influencing traumatic hip dislocation rates. Unlike developed nations, population aging trends are less pronounced, while high-risk behaviors such as widespread motorcycle use without mandatory seatbelt enforcement remain prevalent (29, 30). These factors reduce the impact of interventions that have been effective elsewhere, contributing to slower declines in incidence rates. Delays in patient transportation, limited access to timely diagnostics, and insufficient training at lower-tier hospitals further exacerbate outcomes, with many cases unable to achieve proper reduction within the critical six-hour window, leading to higher rates of femoral head necrosis (30). The elevated EAPC in the ASIR observed in Syria, Yemen, and Libya is strongly correlated with prolonged military conflicts. The compounding effects of battlefield trauma escalation and collapsed healthcare systems have directly precipitated a substantial increase in skeletal injury risks (31, 32). Notably, the concurrent prominence of Afghanistan in both hip dislocation ASIR and age-standardized YLDs metrics underscores how armed conflicts systematically devastate acute trauma care capacity while chronically impairing rehabilitation systems. In contrast, Slovenia’s high incidence rate as a high-income country reveals distinct epidemiological patterns, potentially reflecting the growing public health burden of degenerative joint diseases amid accelerated population aging trends. The pronounced fluctuations in ASIR in low-to-middle SDI regions stem not only from inconsistent enforcement of traffic regulations but also from more aggressive driving behaviors documented among drivers there (33, 34). Likewise, legislators in these regions tend to adopt a less stringent road-safety stance, leading to weaker enforcement and lower sustained compliance with traffic laws (33, 35).

Disability rates differ markedly between regions. In developed countries, the incidence of severe traumatic hip dislocations (Injury Severity Score (ISS) ≥ 9) peaked in 2009 and has since stabilized, with only a marginal annual increase (12). Furthermore, injuries such as sciatic nerve damage are lower than previously reported in other studies, potentially reflecting advancements in treatment and prevention approaches.

An increasing number of orthopedic studies have adopted computational prediction methods—including both traditional statistical models and machine learning techniques—to assess disease trends (36–38). Predictive computational models also highlight demographic shifts, projecting an increase in the number of individuals entering high-risk age groups for hip dislocations. In the present study, the BAPC model demonstrated robust predictive performance. Among males, the rise is predominantly associated with traumatic causes like road injuries and falls, whereas for females, the growing burden of the number of cases and YLDs is driven by age-related factors, such as falls in older populations. These findings underscore the importance of applying computational intelligence to predict future trends, guide intervention strategies, and address disparities in prevention and treatment across different global contexts.

The higher incidence of traumatic hip dislocation in males compared to females is likely multifactorial, primarily attributed to greater exposure to high-energy trauma. This increased exposure stems from men’s higher likelihood of employment in hazardous occupations (e.g., construction, manufacturing), greater participation in high-risk recreational activities and contact sports, and a potential predisposition towards risk-taking behaviors (1, 28). Several studies report a rising incidence of sports-related hip dislocations and its difference in the aspect of gender, however, the GBD 2021 framework does not include a specific category for these injuries (28, 39). While anatomical and biomechanical differences in pelvic structure and muscle mass may play a secondary role, the predominant factor appears to be the increased likelihood of males experiencing the mechanisms that cause traumatic hip dislocation.

Age significantly influences traumatic hip dislocation epidemiology, with distinct patterns observed across different age groups. Young adults (20–39 years) experience the highest incidence, primarily due to high-energy trauma like motor vehicle accidents, often without associated fractures due to stronger bone structure. In contrast, traumatic hip dislocation due to low-energy falls in the older population (65+) is considered to be more common than young adults resulting from age-related declines in bone density and muscle mass, frequently accompanied by fractures and a higher risk of complications like avascular necrosis and post-traumatic arthritis (10, 12, 40). Traumatic hip dislocation is relatively rare in children and adolescents, often associated with the risk of growth plate injuries. The laxity of periacetabular structures has been proposed as a contributing factor in patients under the age of 10, leading to hip dislocations following low-energy trauma (20). These age-related differences reflect variations in bone strength, muscle mass, balance, and the likelihood of comorbidities, influencing both the mechanism of injury and the potential for complications.

### 4.1 Limitations of this study

This study has certain limitations. First, it lacks a more detailed analysis of the subtype of hip dislocation. Second, the analysis did not quantify the quantifiable impact of diagnostic/treatment delays on patient outcomes in developing countries, nor address associated injuries or comorbidities—factors potentially affecting burden assessment accuracy. Third, the availability of health data limits the GBD in 2021. Due to the quality and completeness of the collected information, there may be variations in data accuracy, especially in low-income and underdeveloped areas. This variability may lead to an underestimation or misrepresentation of the health burden in these areas. Fourth, the model used for the 2021 GBD has limitations, which may introduce uncertainties in estimating the disease burden. GBD uncertainty intervals may underestimate the true uncertainty in regions with incomplete data on hip dislocation. Despite these limitations, the 2021 GBD has made significant efforts to address these issues, including updating methods and data sources based on the GBD 2019 to ensure the reliability and robustness of its results. Future versions of the GBD may continue to improve data collection methods and incorporate the latest scientific evidence.

## 5 Conclusion

Hip dislocation continues to pose a significant challenge in terms of disease burden. It not only affects the quality of life of patients but also places a heavy strain on healthcare systems. Data-based frameworks could enhance large-scale policy optimization and clarify future research trajectories. It enables clinicians to make informed decisions, helps policymakers allocate resources effectively, and drives innovation in diagnosis, treatment, and care.

## Data Availability

Publicly available datasets were analyzed in this study. This data can be found at: https://www.healthdata.org/research-analysis/gbd.

## Footnotes

^1^ https://vizhub.healthdata.org/gbd-results/
