# Supplementary figures and images for: A comprehensive study of hip dislocation: global health burden from 1990 to 2021 and its predictions to 2030
Source: Front Public Health. 2025 Sep 9;13:1594523. doi: 10.3389/fpubh.2025.1594523 (PMC12454331; doi:10.3389/fpubh.2025.1594523)

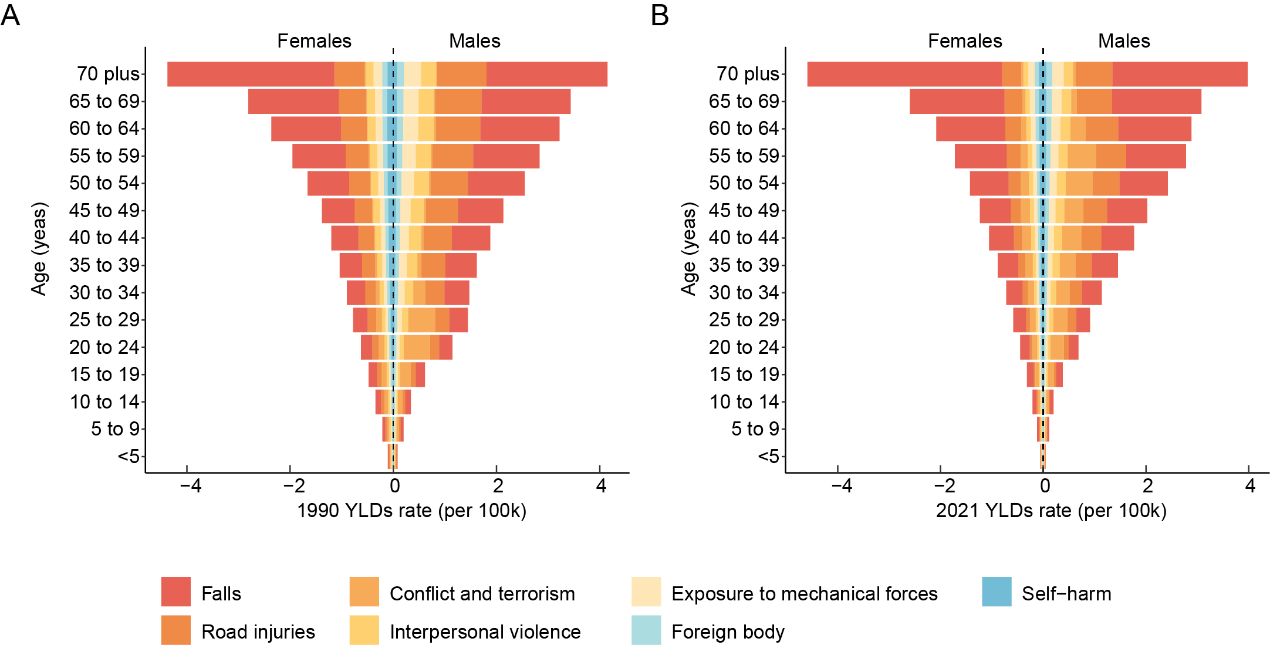

Supplement: Supplementary file 1 [file Image_1.tif]
