# Supplementary material for: A comprehensive study of hip dislocation: global health burden from 1990 to 2021 and its predictions to 2030
Source: Front Public Health. 2025 Sep 9;13:1594523. doi: 10.3389/fpubh.2025.1594523 (PMC12454331; doi:10.3389/fpubh.2025.1594523)
Supplement: Supplementary file 2 [file Image_2.pdf]

Workflow Chart(1)

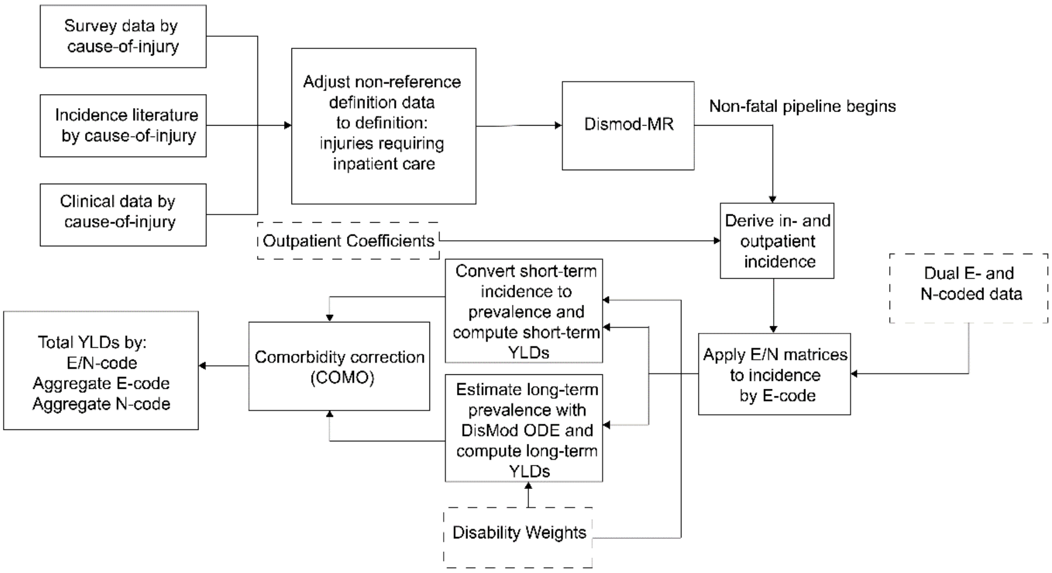

1. Diseases GBD, Injuries C. Global incidence, prevalence, years lived with disability (YLDs), disability-adjusted life-years (DALYs), and healthy life expectancy (HALE) for 371 diseases and injuries in 204 countries and territories and 811 subnational locations, 1990-2021: a systematic analysis for the Global Burden of Disease Study 2021. Lancet. 2024;403(10440):2133-61.
