# Supplementary material for: A comprehensive study of hip dislocation: global health burden from 1990 to 2021 and its predictions to 2030
Source: Front Public Health. 2025 Sep 9;13:1594523. doi: 10.3389/fpubh.2025.1594523 (PMC12454331; doi:10.3389/fpubh.2025.1594523)
Supplement: Supplementary file 3 [file Table_1.docx]

Table S1. Incidence of hip dislocation in 1990 and 2021 for both sexes in countries and territories, with EAPC from 1990 to 2021.

| **Location** | **Number in 1990 (95% UI)** | **Number in 2021 (95% UI)** | **Number change rate (95% UI)** | **CR in 1990 (95% UI)** | **CR in 2021 (95% UI)** | **EAPC of CR, % per year (95% CI)** | **ASR in 1990 (95% UI)** | **ASR in 2021 (95% UI)** | **EAPC of ASR, % per year (95% CI)** |
| --- | --- | --- | --- | --- | --- | --- | --- | --- | --- |
| Afghanistan | 5342 (3657 to 8429) | 54894 (27509 to 113400) | 9.28 (5.8 to 13.55) | 53.72 (36.78 to 84.77) | 175.82 (88.11 to 363.2) | 2.42 (1 to 3.86) | 50.22 (35.16 to 77.77) | 157.41 (78.59 to 322.83) | 2.34 (1 to 3.69) |
| Albania | 2851 (1775 to 4533) | 1661 (1054 to 2586) | -0.42 (-0.48 to -0.33) | 86.24 (53.71 to 137.15) | 62.27 (39.5 to 96.94) | -1.6 (-2.1 to -1.1) | 81.29 (50.58 to 128.3) | 67.87 (42.66 to 106.48) | -1.12 (-1.6 to -0.63) |
| Algeria | 10955 (7366 to 15775) | 13076 (8626 to 19048) | 0.19 (0.06 to 0.35) | 43.32 (29.13 to 62.38) | 29.59 (19.52 to 43.1) | -1.98 (-2.36 to -1.6) | 40.75 (27.34 to 58.26) | 29.65 (19.51 to 43.14) | -1.73 (-2.06 to -1.39) |
| American Samoa | 10 (7 to 15) | 10 (6 to 14) | -0.04 (-0.13 to 0.06) | 21.01 (13.97 to 30.26) | 19.64 (13.01 to 29.12) | -0.04 (-1.14 to 1.08) | 21.49 (14.19 to 31.01) | 20.2 (13.32 to 29.83) | 0.02 (-1.07 to 1.12) |
| Andorra | 19 (12 to 30) | 34 (20 to 56) | 0.77 (0.57 to 0.99) | 35.69 (22.26 to 54.59) | 40.09 (23.74 to 65.95) | 0.34 (0.3 to 0.39) | 35.74 (22.38 to 54.69) | 35.81 (22 to 57.12) | 0.01 (-0.05 to 0.06) |
| Angola | 5862 (3520 to 10639) | 5936 (4194 to 8254) | 0.01 (-0.45 to 0.58) | 57.04 (34.26 to 103.53) | 18.15 (12.82 to 25.24) | -4.56 (-6.05 to -3.05) | 50.48 (31.09 to 90.05) | 18.25 (12.72 to 25.71) | -4.1 (-5.55 to -2.64) |
| Antigua and Barbuda | 19 (13 to 30) | 28 (17 to 46) | 0.47 (0.35 to 0.6) | 31.64 (20.84 to 49.42) | 31.3 (19.43 to 51.27) | -0.32 (-0.81 to 0.18) | 30.58 (20.16 to 47.64) | 32.59 (20.44 to 52.16) | -0.08 (-0.58 to 0.43) |
| Argentina | 12822 (8429 to 19028) | 15034 (9844 to 22026) | 0.17 (0.1 to 0.24) | 38.72 (25.46 to 57.47) | 33.05 (21.64 to 48.42) | -0.49 (-0.66 to -0.32) | 38.59 (25.39 to 57.3) | 33.79 (22.07 to 50.3) | -0.39 (-0.56 to -0.22) |
| Armenia | 1969 (1313 to 2900) | 920 (629 to 1332) | -0.53 (-0.56 to -0.5) | 57.57 (38.37 to 84.77) | 30.7 (21.01 to 44.47) | -1.2 (-2.33 to -0.05) | 56.05 (37.29 to 83.16) | 32.71 (22.35 to 47.85) | -0.83 (-2.05 to 0.4) |
| Australia | 9688 (6184 to 14447) | 12924 (8135 to 19248) | 0.33 (0.22 to 0.49) | 57.47 (36.68 to 85.69) | 50.11 (31.54 to 74.63) | -0.34 (-0.39 to -0.29) | 58.1 (37.34 to 85.8) | 49.57 (31.2 to 73.08) | -0.42 (-0.47 to -0.37) |
| Austria | 3298 (2117 to 5062) | 2974 (1828 to 4622) | -0.1 (-0.19 to -0.03) | 42.45 (27.25 to 65.16) | 33.11 (20.35 to 51.46) | -0.68 (-0.74 to -0.61) | 41.09 (26.18 to 60.43) | 30.96 (19.37 to 46.71) | -0.83 (-0.89 to -0.78) |
| Azerbaijan | 2991 (2037 to 4348) | 3275 (2208 to 4819) | 0.09 (0.02 to 0.16) | 40.83 (27.8 to 59.34) | 31.19 (21.03 to 45.89) | -1.3 (-2.23 to -0.36) | 38.17 (26 to 55.91) | 31.85 (21.55 to 46.77) | -0.97 (-1.94 to 0.01) |
| Bahamas | 66 (46 to 94) | 96 (65 to 140) | 0.44 (0.33 to 0.58) | 25.89 (18.09 to 36.74) | 24.73 (16.82 to 36.13) | 0.26 (-0.32 to 0.84) | 24.8 (17.35 to 35.53) | 25.19 (17.17 to 36.59) | 0.52 (-0.14 to 1.17) |
| Bahrain | 149 (103 to 211) | 402 (266 to 589) | 1.69 (1.42 to 1.98) | 29.49 (20.27 to 41.72) | 26.26 (17.42 to 38.5) | -0.6 (-0.81 to -0.4) | 27.52 (18.82 to 39.19) | 25.57 (16.95 to 37.5) | -0.49 (-0.67 to -0.31) |
| Bangladesh | 19951 (14220 to 27324) | 26587 (17536 to 38852) | 0.33 (0.13 to 0.54) | 18.28 (13.03 to 25.04) | 16.15 (10.65 to 23.6) | -1.28 (-2.55 to 0.01) | 16.73 (11.7 to 23.26) | 15.81 (10.52 to 23.05) | -1.01 (-2.23 to 0.22) |
| Barbados | 57 (40 to 85) | 64 (43 to 96) | 0.12 (0.02 to 0.23) | 22.67 (15.63 to 33.37) | 21.47 (14.38 to 32.16) | -0.43 (-0.59 to -0.27) | 22.56 (15.54 to 33.21) | 23.13 (15.67 to 34.39) | -0.15 (-0.32 to 0.01) |
| Belarus | 7361 (4718 to 11088) | 6193 (3899 to 9605) | -0.16 (-0.24 to -0.07) | 70.48 (45.18 to 106.16) | 66.42 (41.81 to 103.01) | -0.25 (-0.6 to 0.1) | 71.88 (45.71 to 108.45) | 67.94 (42.67 to 105.14) | -0.28 (-0.57 to 0.01) |
| Belgium | 3931 (2566 to 5801) | 4583 (2784 to 7277) | 0.17 (0.02 to 0.31) | 39.4 (25.72 to 58.14) | 39.96 (24.27 to 63.45) | 0.28 (-0.02 to 0.59) | 37.99 (24.96 to 55.46) | 34.44 (21.22 to 53.67) | -0.08 (-0.48 to 0.31) |
| Belize | 68 (48 to 97) | 144 (98 to 213) | 1.11 (0.74 to 1.43) | 36.43 (25.55 to 51.97) | 33.54 (22.75 to 49.65) | -0.34 (-0.68 to 0.01) | 31.95 (22.53 to 45.77) | 31.99 (21.58 to 46.99) | -0.18 (-0.5 to 0.14) |
| Benin | 1027 (737 to 1422) | 2572 (1824 to 3530) | 1.51 (1.36 to 1.67) | 21.17 (15.19 to 29.32) | 19.05 (13.51 to 26.15) | -0.41 (-0.47 to -0.35) | 19.5 (13.7 to 27.19) | 18.78 (13 to 26.44) | -0.21 (-0.26 to -0.15) |
| Bermuda | 15 (10 to 21) | 15 (10 to 23) | 0.01 (-0.12 to 0.18) | 24.61 (16.76 to 36.12) | 23.2 (15.16 to 35.6) | -0.25 (-0.36 to -0.14) | 25.28 (17.32 to 37.39) | 25 (16.51 to 37.59) | -0.1 (-0.24 to 0.04) |
| Bhutan | 138 (93 to 199) | 202 (131 to 304) | 0.46 (0.25 to 0.74) | 21.89 (14.73 to 31.6) | 26.69 (17.34 to 40.21) | 0.19 (-0.44 to 0.81) | 21.91 (14.64 to 31.81) | 27.61 (18.02 to 41.67) | 0.31 (-0.28 to 0.9) |
| Bolivia (Plurinational State of) | 2179 (1553 to 3036) | 3364 (2311 to 4861) | 0.54 (0.39 to 0.7) | 34.15 (24.34 to 47.58) | 28.51 (19.59 to 41.21) | -0.66 (-0.71 to -0.61) | 32.97 (23.3 to 46.08) | 28.5 (19.56 to 41.09) | -0.54 (-0.59 to -0.49) |
| Bosnia and Herzegovina | 4085 (2611 to 6169) | 1731 (1086 to 2714) | -0.58 (-0.66 to -0.48) | 90.82 (58.04 to 137.15) | 52.41 (32.89 to 82.17) | -3.33 (-4.6 to -2.03) | 90.76 (57.94 to 137.38) | 58.58 (36.78 to 91.62) | -3.03 (-4.33 to -1.72) |
| Botswana | 280 (197 to 385) | 522 (365 to 732) | 0.87 (0.69 to 1.06) | 21.2 (14.97 to 29.22) | 21.82 (15.25 to 30.59) | 0.05 (-0.13 to 0.23) | 20.64 (14.41 to 28.81) | 21.43 (14.94 to 30.06) | 0.02 (-0.14 to 0.19) |
| Brazil | 79145 (52257 to 118159) | 90319 (59635 to 134377) | 0.14 (0.08 to 0.22) | 53.29 (35.19 to 79.56) | 40.99 (27.06 to 60.98) | -0.65 (-0.79 to -0.51) | 51.9 (34.26 to 77.27) | 40.47 (26.86 to 60.07) | -0.54 (-0.68 to -0.41) |
| Brunei Darussalam | 94 (62 to 131) | 130 (84 to 189) | 0.39 (0.28 to 0.49) | 36.29 (23.96 to 50.5) | 28.92 (18.68 to 41.85) | -0.73 (-0.78 to -0.68) | 35.97 (23.8 to 49.39) | 29.04 (18.87 to 41.86) | -0.69 (-0.73 to -0.65) |
| Bulgaria | 7137 (4559 to 10804) | 4259 (2601 to 6716) | -0.4 (-0.46 to -0.34) | 82.23 (52.52 to 124.47) | 62.75 (38.33 to 98.95) | -0.97 (-1.01 to -0.93) | 87.56 (56.55 to 129.84) | 72.04 (44.94 to 112.38) | -0.68 (-0.7 to -0.65) |
| Burkina Faso | 1996 (1408 to 2777) | 6171 (4263 to 8756) | 2.09 (1.52 to 3.52) | 20.94 (14.78 to 29.14) | 27.11 (18.73 to 38.47) | 0.48 (0.08 to 0.88) | 19.81 (13.72 to 27.94) | 25.73 (18.11 to 35.85) | 0.55 (0.2 to 0.89) |
| Burundi | 1163 (829 to 1636) | 2565 (1850 to 3449) | 1.21 (1.02 to 1.55) | 20.94 (14.92 to 29.45) | 19.4 (13.99 to 26.09) | -6.27 (-9.65 to -2.76) | 20.12 (14.28 to 28.16) | 18.87 (13.49 to 25.88) | -6.01 (-9.24 to -2.67) |
| Cabo Verde | 76 (55 to 108) | 109 (75 to 158) | 0.43 (0.26 to 0.62) | 21.55 (15.44 to 30.6) | 19.52 (13.43 to 28.26) | -0.25 (-0.3 to -0.2) | 20.35 (14.41 to 28.6) | 19.87 (13.63 to 28.8) | -0.02 (-0.07 to 0.03) |
| Cambodia | 3515 (2427 to 5138) | 4393 (2830 to 6701) | 0.25 (-0.16 to 0.67) | 34.21 (23.63 to 50.01) | 25.78 (16.6 to 39.32) | -1.22 (-1.75 to -0.7) | 33.17 (22.87 to 47.19) | 26.7 (17.27 to 41.39) | -0.97 (-1.44 to -0.5) |
| Cameroon | 1944 (1369 to 2665) | 7013 (5011 to 9646) | 2.61 (2.14 to 3.57) | 18.63 (13.12 to 25.53) | 22.07 (15.77 to 30.35) | 0.89 (0.58 to 1.21) | 18.2 (12.68 to 25.87) | 21.67 (15.27 to 30.01) | 0.83 (0.57 to 1.1) |
| Canada | 8400 (5558 to 11731) | 10951 (7053 to 16446) | 0.3 (0.12 to 0.51) | 30.82 (20.39 to 43.04) | 29.23 (18.82 to 43.89) | -0.04 (-0.09 to 0.01) | 29.55 (19.49 to 41.31) | 24.28 (15.8 to 34.86) | -0.55 (-0.59 to -0.52) |
| Central African Republic | 596 (428 to 835) | 2807 (1835 to 4685) | 3.71 (2.14 to 7.29) | 21.82 (15.68 to 30.58) | 51.19 (33.46 to 85.42) | 2.88 (1.68 to 4.1) | 20.89 (14.88 to 29.09) | 45.12 (30.04 to 71.91) | 2.63 (1.52 to 3.75) |
| Chad | 2085 (1411 to 3331) | 4768 (3335 to 6733) | 1.29 (0.95 to 1.58) | 34.6 (23.41 to 55.28) | 26.86 (18.79 to 37.93) | -0.92 (-1.78 to -0.04) | 30.93 (20.88 to 48.66) | 24.7 (17.59 to 34.04) | -0.77 (-1.58 to 0.05) |
| Chile | 4668 (3040 to 6755) | 6328 (3931 to 9811) | 0.36 (0.23 to 0.48) | 35.13 (22.88 to 50.85) | 33.66 (20.91 to 52.19) | -0.02 (-0.19 to 0.14) | 34.45 (22.43 to 49.94) | 35.18 (21.99 to 53.52) | 0.22 (0.05 to 0.4) |
| China | 311602 (208484 to 452304) | 410680 (254091 to 649132) | 0.32 (0.14 to 0.52) | 26.49 (17.72 to 38.45) | 28.87 (17.86 to 45.63) | 0 (-0.32 to 0.32) | 26.58 (17.65 to 38.88) | 27.56 (17.35 to 43.16) | -0.25 (-0.58 to 0.08) |
| Colombia | 19189 (13065 to 27698) | 15909 (10478 to 23148) | -0.17 (-0.3 to -0.08) | 59.06 (40.21 to 85.25) | 32.43 (21.36 to 47.18) | -2.24 (-2.45 to -2.02) | 54.77 (37.21 to 78.21) | 33.81 (22.24 to 48.97) | -1.82 (-2.01 to -1.63) |
| Comoros | 93 (66 to 129) | 126 (88 to 178) | 0.35 (0.25 to 0.46) | 20.16 (14.22 to 27.88) | 16.93 (11.82 to 23.96) | -0.13 (-0.74 to 0.48) | 19.39 (13.78 to 26.94) | 17.24 (12.05 to 24.45) | 0.02 (-0.52 to 0.57) |
| Congo | 479 (339 to 664) | 892 (639 to 1224) | 0.86 (0.75 to 1.01) | 19.93 (14.1 to 27.63) | 16.55 (11.85 to 22.7) | -2.7 (-4.88 to -0.46) | 19.82 (13.79 to 27.77) | 17.06 (11.96 to 24.01) | -2.41 (-4.45 to -0.33) |
| Cook Islands | 5 (3 to 7) | 4 (2 to 5) | -0.25 (-0.46 to -0.05) | 25.04 (17.22 to 34.82) | 19.98 (12.74 to 30.32) | -0.58 (-1.56 to 0.42) | 24.92 (17.14 to 34.5) | 19.94 (12.86 to 30.33) | -0.56 (-1.55 to 0.45) |
| Costa Rica | 1205 (782 to 1840) | 1573 (1023 to 2391) | 0.3 (0.2 to 0.41) | 39.63 (25.71 to 60.51) | 33.12 (21.55 to 50.35) | -0.66 (-0.7 to -0.61) | 38.15 (24.65 to 58.06) | 34.53 (22.3 to 53) | -0.35 (-0.4 to -0.29) |
| Côte d'Ivoire | 2398 (1713 to 3322) | 5031 (3539 to 7077) | 1.1 (0.97 to 1.24) | 19.66 (14.04 to 27.24) | 18.06 (12.7 to 25.4) | -0.24 (-0.67 to 0.19) | 19.36 (13.61 to 26.92) | 18.54 (12.9 to 26.49) | -0.15 (-0.52 to 0.22) |
| Croatia | 4153 (2720 to 6184) | 3235 (1922 to 5311) | -0.22 (-0.39 to -0.03) | 85.43 (55.96 to 127.21) | 76.86 (45.66 to 126.2) | -1.17 (-1.72 to -0.62) | 86.53 (57.21 to 127.81) | 67.26 (41.44 to 106.65) | -1.75 (-2.32 to -1.18) |
| Cuba | 3857 (2603 to 5684) | 4796 (2994 to 7672) | 0.24 (0.03 to 0.51) | 35.56 (23.99 to 52.4) | 42.55 (26.57 to 68.08) | 0.54 (0.5 to 0.58) | 35.03 (23.68 to 52.12) | 38.58 (25.09 to 59.32) | 0.27 (0.17 to 0.37) |
| Cyprus | 280 (184 to 397) | 416 (265 to 628) | 0.48 (0.32 to 0.66) | 36.04 (23.62 to 51.08) | 30.64 (19.5 to 46.28) | -0.56 (-0.68 to -0.43) | 36.56 (23.94 to 51.89) | 30.22 (19.29 to 44.73) | -0.62 (-0.74 to -0.51) |
| Czechia | 10704 (6797 to 16784) | 7443 (4582 to 11946) | -0.3 (-0.35 to -0.26) | 103.98 (66.02 to 163.04) | 70.01 (43.09 to 112.36) | -1.34 (-1.48 to -1.19) | 102.16 (65.73 to 158.13) | 70.58 (44.16 to 110.74) | -1.11 (-1.29 to -0.93) |
| Democratic People's Republic of Korea | 3981 (2729 to 5742) | 4440 (3085 to 6523) | 0.12 (0.02 to 0.22) | 19.33 (13.25 to 27.89) | 16.82 (11.69 to 24.72) | -0.33 (-0.44 to -0.22) | 19.04 (13.04 to 27.53) | 16 (11.14 to 23.05) | -0.46 (-0.58 to -0.34) |
| Democratic Republic of the Congo | 8468 (5951 to 11712) | 20709 (14879 to 28592) | 1.45 (1.12 to 2.09) | 22.2 (15.6 to 30.7) | 23.01 (16.53 to 31.76) | -1.18 (-2.52 to 0.17) | 20.41 (14.43 to 28.41) | 22.22 (15.99 to 29.77) | -0.95 (-2.22 to 0.33) |
| Denmark | 2059 (1320 to 3013) | 1618 (1010 to 2479) | -0.21 (-0.28 to -0.16) | 40.02 (25.66 to 58.57) | 27.64 (17.26 to 42.37) | -1.3 (-1.36 to -1.23) | 35.9 (23.28 to 52.49) | 25.98 (16.42 to 39.02) | -1.06 (-1.11 to -1) |
| Djibouti | 151 (99 to 248) | 218 (154 to 301) | 0.45 (-0.16 to 1.05) | 36.44 (23.84 to 59.86) | 17.35 (12.26 to 23.9) | -2.19 (-2.91 to -1.46) | 32.18 (22.18 to 50.99) | 18.01 (12.68 to 25.02) | -1.73 (-2.36 to -1.09) |
| Dominica | 17 (12 to 25) | 15 (10 to 22) | -0.11 (-0.17 to -0.04) | 23.85 (16.75 to 33.86) | 22.92 (15.6 to 33.11) | 0.43 (-0.24 to 1.09) | 22.85 (16.11 to 32.52) | 23.55 (16.01 to 34.25) | 0.71 (-0.02 to 1.44) |
| Dominican Republic | 1837 (1299 to 2599) | 3120 (2148 to 4504) | 0.7 (0.57 to 0.81) | 25.69 (18.16 to 36.35) | 28.33 (19.51 to 40.9) | 0.33 (0.13 to 0.54) | 23.99 (17.06 to 33.98) | 27.96 (19.29 to 40.45) | 0.51 (0.29 to 0.72) |
| Ecuador | 3498 (2404 to 4986) | 6138 (4095 to 9040) | 0.75 (0.62 to 0.89) | 35.06 (24.1 to 49.97) | 33.97 (22.67 to 50.04) | -0.18 (-0.34 to -0.03) | 33.89 (23.17 to 48.35) | 33.5 (22.36 to 49.28) | -0.1 (-0.25 to 0.05) |
| Egypt | 21457 (14595 to 29969) | 29630 (20697 to 41410) | 0.38 (0.22 to 0.56) | 38.78 (26.38 to 54.16) | 28.05 (19.59 to 39.2) | -0.77 (-0.93 to -0.62) | 36.17 (24.6 to 50.77) | 27.92 (19.39 to 39.09) | -0.59 (-0.73 to -0.45) |
| El Salvador | 4639 (2949 to 7581) | 2412 (1615 to 3505) | -0.48 (-0.7 to -0.23) | 87.43 (55.58 to 142.89) | 37.4 (25.03 to 54.34) | -1.34 (-1.93 to -0.75) | 75.31 (48.84 to 122.28) | 36.96 (24.73 to 53.56) | -1.02 (-1.56 to -0.47) |
| Equatorial Guinea | 93 (66 to 129) | 235 (163 to 326) | 1.54 (1.27 to 1.87) | 21.9 (15.52 to 30.48) | 15.54 (10.75 to 21.59) | -1.2 (-1.29 to -1.1) | 20.57 (14.75 to 28.7) | 16.15 (11.15 to 23.18) | -0.83 (-0.91 to -0.76) |
| Eritrea | 9688 (4349 to 21647) | 1216 (861 to 1696) | -0.87 (-0.95 to -0.71) | 284.44 (127.68 to 635.58) | 18.43 (13.06 to 25.71) | -4.18 (-6.59 to -1.7) | 241.83 (110.11 to 540.04) | 19.09 (13.43 to 26.76) | -3.77 (-6.05 to -1.44) |
| Estonia | 1379 (894 to 2050) | 675 (433 to 1056) | -0.51 (-0.55 to -0.48) | 87.94 (57.01 to 130.68) | 51.51 (33.04 to 80.52) | -2.07 (-2.21 to -1.93) | 89.23 (57.54 to 132.81) | 54.42 (34.78 to 85.65) | -1.93 (-2.04 to -1.81) |
| Eswatini | 177 (128 to 242) | 355 (247 to 494) | 1 (0.59 to 1.86) | 21.96 (15.87 to 30.05) | 30.69 (21.38 to 42.77) | 0.34 (0.12 to 0.56) | 21.49 (15.39 to 29.7) | 28.61 (20.12 to 38.91) | 0.25 (0.05 to 0.46) |
| Ethiopia | 68965 (35114 to 142181) | 34813 (21779 to 62356) | -0.5 (-0.67 to -0.22) | 136.38 (69.44 to 281.16) | 31.96 (19.99 to 57.24) | -2.76 (-4.3 to -1.18) | 119.84 (62.43 to 243.72) | 29.45 (19.18 to 49.34) | -2.59 (-3.99 to -1.17) |
| Fiji | 129 (87 to 183) | 143 (96 to 204) | 0.11 (0.03 to 0.19) | 17 (11.51 to 24.19) | 15.43 (10.42 to 22.04) | -0.51 (-0.78 to -0.25) | 16.74 (11.2 to 23.8) | 15.7 (10.61 to 22.53) | -0.4 (-0.66 to -0.13) |
| Finland | 2513 (1630 to 3679) | 2287 (1395 to 3597) | -0.09 (-0.2 to 0.01) | 50.16 (32.54 to 73.42) | 41.31 (25.2 to 64.98) | -0.7 (-1.17 to -0.23) | 47.68 (31.03 to 69.84) | 36.75 (22.64 to 57.14) | -0.91 (-1.41 to -0.4) |
| France | 26371 (17272 to 38445) | 26373 (16497 to 40459) | 0 (-0.1 to 0.1) | 45.65 (29.9 to 66.55) | 39.72 (24.85 to 60.94) | -0.37 (-0.42 to -0.33) | 42.45 (27.83 to 61.64) | 33.75 (21.26 to 51.58) | -0.67 (-0.72 to -0.61) |
| Gabon | 207 (145 to 285) | 326 (227 to 457) | 0.58 (0.48 to 0.68) | 21.06 (14.77 to 28.95) | 17.97 (12.52 to 25.16) | -0.54 (-0.61 to -0.48) | 20.94 (14.64 to 29.31) | 18.59 (13.01 to 26.53) | -0.44 (-0.49 to -0.38) |
| Gambia | 176 (123 to 246) | 390 (273 to 555) | 1.21 (1.05 to 1.4) | 17.96 (12.53 to 25.1) | 16.28 (11.4 to 23.18) | -0.37 (-0.55 to -0.2) | 17 (11.81 to 24.11) | 16.87 (11.59 to 24.65) | -0.1 (-0.24 to 0.04) |
| Georgia | 3002 (1925 to 4524) | 1935 (1234 to 2966) | -0.36 (-0.39 to -0.32) | 54.35 (34.85 to 81.9) | 53.65 (34.21 to 82.22) | -0.31 (-0.87 to 0.25) | 55.26 (35.43 to 82.98) | 58 (36.98 to 88.8) | -0.15 (-0.72 to 0.42) |
| Germany | 26835 (17543 to 39498) | 26758 (16504 to 41588) | 0 (-0.11 to 0.08) | 33.57 (21.94 to 49.41) | 31.34 (19.33 to 48.71) | -0.22 (-0.3 to -0.13) | 32.69 (21.62 to 47.91) | 27.94 (17.51 to 42.02) | -0.54 (-0.57 to -0.51) |
| Ghana | 2655 (1859 to 3691) | 5778 (4001 to 8212) | 1.18 (1.02 to 1.35) | 17.73 (12.42 to 24.65) | 16.87 (11.68 to 23.98) | -0.17 (-0.27 to -0.08) | 17.38 (12.12 to 24.72) | 17.37 (11.93 to 24.98) | -0.04 (-0.13 to 0.05) |
| Greece | 3623 (2395 to 5190) | 2659 (1753 to 3793) | -0.27 (-0.32 to -0.21) | 34.87 (23.05 to 49.96) | 26.13 (17.23 to 37.28) | -0.98 (-1.03 to -0.92) | 35.17 (23.45 to 50.92) | 26.29 (16.85 to 38.32) | -0.96 (-1.04 to -0.88) |
| Greenland | 23 (15 to 32) | 19 (12 to 28) | -0.16 (-0.26 to -0.06) | 40.93 (27.16 to 58.48) | 33.86 (21.94 to 50.2) | -0.7 (-0.81 to -0.6) | 41.99 (28.18 to 59.82) | 31.67 (20.89 to 46.91) | -1.06 (-1.17 to -0.96) |
| Grenada | 25 (17 to 37) | 30 (19 to 46) | 0.21 (0.09 to 0.32) | 28.78 (19.8 to 42.62) | 29.43 (18.98 to 45.22) | -0.02 (-0.26 to 0.22) | 27.97 (19.2 to 41.3) | 29.7 (19.39 to 45.49) | 0.13 (-0.11 to 0.37) |
| Guam | 26 (18 to 39) | 28 (18 to 43) | 0.06 (-0.06 to 0.17) | 19.29 (12.81 to 28.2) | 17.5 (11.49 to 26.75) | -0.31 (-0.51 to -0.12) | 18.93 (12.5 to 27.95) | 17.58 (11.52 to 26.65) | -0.23 (-0.43 to -0.03) |
| Guatemala | 5683 (3806 to 8477) | 7172 (4626 to 10727) | 0.26 (-0.18 to 0.63) | 67.78 (45.39 to 101.1) | 45.49 (29.34 to 68.03) | -0.7 (-0.96 to -0.45) | 59.98 (39.89 to 88.18) | 44.29 (28.78 to 66.55) | -0.41 (-0.64 to -0.18) |
| Guinea | 1286 (915 to 1764) | 2590 (1830 to 3559) | 1.01 (0.9 to 1.14) | 21.46 (15.26 to 29.43) | 19.29 (13.63 to 26.5) | -0.32 (-0.88 to 0.23) | 19.32 (13.56 to 26.66) | 18.8 (13.11 to 26.13) | -0.09 (-0.6 to 0.42) |
| Guinea-Bissau | 243 (171 to 336) | 392 (275 to 545) | 0.61 (0.53 to 0.71) | 24.09 (17.01 to 33.34) | 18.97 (13.34 to 26.42) | -1.31 (-2.27 to -0.34) | 22.91 (16.05 to 31.9) | 19.65 (13.67 to 27.73) | -0.98 (-1.85 to -0.12) |
| Guyana | 239 (170 to 334) | 263 (180 to 373) | 0.1 (0.01 to 0.19) | 30.64 (21.75 to 42.86) | 34.34 (23.58 to 48.81) | 0.25 (-0.02 to 0.52) | 29.75 (20.88 to 41.98) | 34.06 (23.35 to 48.43) | 0.31 (0.05 to 0.57) |
| Haiti | 2142 (1506 to 3051) | 5892 (3940 to 8676) | 1.75 (1.2 to 3.02) | 33.56 (23.61 to 47.81) | 45.8 (30.63 to 67.44) | 0.18 (-1.77 to 2.16) | 31.58 (22.13 to 44.71) | 43.78 (29.25 to 64.7) | 0.27 (-1.7 to 2.29) |
| Honduras | 2066 (1366 to 3036) | 3448 (2313 to 5064) | 0.67 (0.55 to 0.83) | 43.87 (28.99 to 64.47) | 34.1 (22.88 to 50.09) | -1.26 (-2.13 to -0.38) | 38.22 (25.49 to 56.01) | 33.25 (22.45 to 48.19) | -0.89 (-1.75 to -0.02) |
| Hungary | 10098 (6302 to 16124) | 6407 (3934 to 10402) | -0.37 (-0.4 to -0.34) | 97.14 (60.63 to 155.12) | 66.75 (40.99 to 108.38) | -1.54 (-1.67 to -1.4) | 93 (58.93 to 143.04) | 64.77 (40.39 to 100.84) | -1.4 (-1.51 to -1.29) |
| Iceland | 87 (56 to 125) | 99 (62 to 152) | 0.14 (0.05 to 0.24) | 34.08 (22.07 to 49.12) | 28.27 (17.78 to 43.25) | -0.83 (-1.31 to -0.34) | 33.55 (21.53 to 48.17) | 27.64 (17.41 to 41.78) | -0.86 (-1.36 to -0.36) |
| India | 312341 (208680 to 450285) | 434893 (271390 to 676027) | 0.39 (0.16 to 0.59) | 36.61 (24.46 to 52.78) | 30.75 (19.19 to 47.79) | -0.64 (-0.74 to -0.54) | 38.83 (25.59 to 56.81) | 31.67 (19.67 to 50) | -0.74 (-0.82 to -0.65) |
| Indonesia | 50959 (34264 to 73220) | 54031 (35985 to 79088) | 0.06 (-0.05 to 0.19) | 27.55 (18.52 to 39.58) | 19.37 (12.9 to 28.36) | -1.26 (-1.61 to -0.91) | 27.54 (18.41 to 39.62) | 20.03 (13.4 to 29.61) | -1.15 (-1.49 to -0.8) |
| Iran (Islamic Republic of) | 39095 (26547 to 55998) | 27772 (18749 to 40113) | -0.29 (-0.51 to -0.09) | 68.47 (46.49 to 98.07) | 32.54 (21.97 to 47) | -1.62 (-1.88 to -1.35) | 64.77 (44.59 to 91.69) | 32.68 (22.05 to 47.16) | -1.47 (-1.73 to -1.21) |
| Iraq | 11747 (8513 to 16598) | 18098 (12805 to 25567) | 0.54 (0.33 to 0.72) | 63.78 (46.22 to 90.12) | 43.9 (31.06 to 62.02) | 1.48 (-0.24 to 3.22) | 62.4 (45.84 to 87.86) | 42.87 (30.4 to 59.99) | 1.35 (-0.29 to 3.01) |
| Ireland | 1119 (722 to 1617) | 1344 (835 to 2052) | 0.2 (0.07 to 0.32) | 31.07 (20.06 to 44.89) | 27.21 (16.89 to 41.52) | -0.48 (-0.6 to -0.36) | 31.04 (19.95 to 44.75) | 27.27 (17.15 to 41.18) | -0.43 (-0.56 to -0.31) |
| Israel | 1665 (1091 to 2330) | 2942 (1909 to 4095) | 0.77 (0.61 to 0.97) | 33.56 (21.99 to 46.97) | 30.66 (19.9 to 42.68) | -0.57 (-1.44 to 0.31) | 33.11 (21.74 to 46.39) | 30.51 (19.92 to 42.92) | -0.53 (-1.42 to 0.36) |
| Italy | 23303 (14813 to 35356) | 17518 (10604 to 27675) | -0.25 (-0.32 to -0.18) | 41.03 (26.08 to 62.25) | 29.29 (17.73 to 46.27) | -1.33 (-1.44 to -1.22) | 39.38 (25.37 to 58.81) | 27.47 (16.9 to 42.49) | -1.38 (-1.5 to -1.26) |
| Jamaica | 746 (507 to 1121) | 843 (575 to 1251) | 0.13 (0.01 to 0.29) | 31.55 (21.45 to 47.4) | 30.12 (20.52 to 44.67) | -0.31 (-0.53 to -0.08) | 29.76 (20.07 to 45.38) | 30.28 (20.7 to 44.81) | -0.11 (-0.33 to 0.1) |
| Japan | 39671 (25493 to 56879) | 31154 (20152 to 44198) | -0.21 (-0.29 to -0.13) | 31.53 (20.26 to 45.2) | 24.4 (15.78 to 34.61) | -0.88 (-1.07 to -0.7) | 31.94 (20.62 to 45.21) | 23.47 (15.12 to 33.94) | -1.03 (-1.23 to -0.82) |
| Jordan | 1191 (815 to 1681) | 3019 (1995 to 4435) | 1.53 (1.28 to 1.84) | 31.89 (21.82 to 44.99) | 24.49 (16.18 to 35.98) | -0.83 (-0.93 to -0.73) | 29.65 (20.31 to 41.84) | 23.54 (15.51 to 34.76) | -0.71 (-0.79 to -0.63) |
| Kazakhstan | 9364 (6179 to 13762) | 8865 (5867 to 13405) | -0.05 (-0.1 to 0) | 57.12 (37.69 to 83.95) | 46.77 (30.95 to 70.72) | -0.56 (-0.72 to -0.41) | 55.02 (36.23 to 81.24) | 46.86 (30.94 to 70.76) | -0.47 (-0.59 to -0.35) |
| Kenya | 4520 (3207 to 6323) | 8557 (6073 to 12022) | 0.89 (0.73 to 1.05) | 19.53 (13.86 to 27.32) | 17.09 (12.13 to 24.01) | -0.48 (-0.79 to -0.16) | 19.47 (13.59 to 27.4) | 18.29 (12.68 to 25.72) | -0.24 (-0.5 to 0.03) |
| Kiribati | 11 (8 to 16) | 15 (11 to 22) | 0.37 (0.23 to 0.49) | 15.08 (10.62 to 21.63) | 12.7 (8.72 to 18.04) | -1.26 (-1.92 to -0.6) | 14.74 (10.3 to 21.13) | 12.57 (8.63 to 17.94) | -1.2 (-1.8 to -0.59) |
| Kuwait | 2078 (1166 to 3888) | 1435 (919 to 2198) | -0.31 (-0.67 to 0.26) | 120.9 (67.84 to 226.23) | 30.85 (19.77 to 47.27) | -2.14 (-3.25 to -1.02) | 115.2 (64.08 to 219.63) | 29.6 (19.06 to 45.01) | -2.09 (-3.24 to -0.92) |
| Kyrgyzstan | 2248 (1501 to 3294) | 2372 (1606 to 3397) | 0.06 (-0.01 to 0.15) | 50.35 (33.62 to 73.79) | 34.56 (23.4 to 49.49) | -1.45 (-1.66 to -1.23) | 47.86 (32.26 to 70.71) | 33.52 (22.74 to 47.96) | -1.38 (-1.57 to -1.19) |
| Lao People's Democratic Republic | 1648 (1059 to 2841) | 1409 (959 to 2005) | -0.15 (-0.53 to 0.23) | 39.53 (25.41 to 68.14) | 19.09 (13 to 27.17) | -0.92 (-1.27 to -0.57) | 37.31 (24.4 to 61.54) | 19.14 (12.92 to 27.47) | -0.89 (-1.21 to -0.58) |
| Latvia | 2599 (1673 to 3889) | 1056 (681 to 1644) | -0.59 (-0.62 to -0.57) | 97.77 (62.94 to 146.3) | 56.48 (36.4 to 87.92) | -2.28 (-2.48 to -2.07) | 98.25 (62.71 to 147.1) | 58.6 (37.28 to 91.91) | -2.15 (-2.33 to -1.97) |
| Lebanon | 2219 (1373 to 3845) | 1477 (980 to 2120) | -0.33 (-0.62 to 0.03) | 74.18 (45.88 to 128.5) | 26.65 (17.69 to 38.26) | -1.75 (-2.47 to -1.02) | 71.7 (44.55 to 120.42) | 26.38 (17.51 to 37.61) | -1.86 (-2.57 to -1.15) |
| Lesotho | 309 (223 to 424) | 465 (338 to 653) | 0.5 (0.34 to 0.65) | 20.18 (14.56 to 27.68) | 24.8 (18.05 to 34.82) | 0.69 (0.41 to 0.96) | 19.58 (14.16 to 27.43) | 24.15 (17.57 to 33.98) | 0.72 (0.47 to 0.97) |
| Liberia | 4161 (1958 to 9019) | 858 (594 to 1207) | -0.79 (-0.91 to -0.55) | 169.09 (79.58 to 366.52) | 15.71 (10.88 to 22.11) | -6.45 (-8.53 to -4.32) | 148.7 (71 to 322.22) | 16.08 (11.17 to 23.28) | -5.96 (-7.99 to -3.89) |
| Libya | 1523 (1023 to 2209) | 2517 (1737 to 3574) | 0.65 (0.43 to 0.99) | 36.14 (24.26 to 52.41) | 36.63 (25.28 to 52.03) | 2.43 (0.97 to 3.92) | 34.72 (23.38 to 50.34) | 38.05 (26.48 to 54.91) | 2.73 (1.3 to 4.18) |
| Lithuania | 3236 (2067 to 4830) | 1661 (1035 to 2577) | -0.49 (-0.53 to -0.44) | 88.08 (56.25 to 131.46) | 60.86 (37.94 to 94.45) | -1.42 (-1.62 to -1.21) | 87.56 (55.73 to 130.17) | 60.28 (38.3 to 93.56) | -1.42 (-1.61 to -1.22) |
| Luxembourg | 187 (121 to 263) | 220 (138 to 335) | 0.18 (0.02 to 0.34) | 49.03 (31.75 to 69.08) | 34.2 (21.4 to 52.05) | -1.16 (-1.27 to -1.06) | 46.24 (30.5 to 66.21) | 32.18 (20.21 to 48.94) | -1.21 (-1.3 to -1.12) |
| Madagascar | 2381 (1674 to 3356) | 4717 (3396 to 6504) | 0.98 (0.86 to 1.19) | 20.01 (14.07 to 28.2) | 16.52 (11.89 to 22.78) | -0.61 (-0.73 to -0.5) | 18.39 (13.05 to 25.99) | 16.04 (11.27 to 22.35) | -0.46 (-0.56 to -0.37) |
| Malawi | 1885 (1313 to 2643) | 3094 (2147 to 4308) | 0.64 (0.52 to 0.78) | 19.22 (13.39 to 26.95) | 15.91 (11.04 to 22.15) | -0.67 (-0.77 to -0.58) | 17.55 (12.12 to 24.67) | 15.88 (10.84 to 22.74) | -0.4 (-0.48 to -0.32) |
| Malaysia | 3713 (2546 to 5250) | 6727 (4526 to 9790) | 0.81 (0.63 to 1.01) | 21.02 (14.41 to 29.72) | 21.15 (14.23 to 30.77) | -0.06 (-0.11 to 0) | 21.54 (14.66 to 30.6) | 20.9 (14.26 to 30.39) | -0.19 (-0.23 to -0.14) |
| Maldives | 54 (36 to 79) | 114 (74 to 174) | 1.11 (0.72 to 1.55) | 24.32 (16.37 to 35.62) | 22.02 (14.27 to 33.71) | -0.24 (-1.02 to 0.55) | 24.88 (16.81 to 36.69) | 21.65 (14.04 to 33.13) | -0.45 (-1.24 to 0.34) |
| Mali | 2687 (1884 to 3869) | 6064 (4301 to 8316) | 1.26 (1.02 to 1.47) | 31.01 (21.74 to 44.66) | 25.16 (17.84 to 34.5) | -1.24 (-2.78 to 0.32) | 28.44 (20.03 to 40.32) | 23.68 (16.81 to 32.6) | -1.14 (-2.6 to 0.34) |
| Malta | 127 (81 to 186) | 134 (84 to 204) | 0.06 (-0.02 to 0.15) | 34.16 (21.99 to 50.12) | 30.41 (18.93 to 46.12) | -0.35 (-0.41 to -0.29) | 34.51 (22.33 to 50.82) | 29.6 (18.56 to 44.94) | -0.42 (-0.53 to -0.31) |
| Marshall Islands | 9 (6 to 12) | 11 (7 to 15) | 0.25 (0.15 to 0.36) | 18.91 (12.87 to 26.46) | 19.07 (12.85 to 27.54) | 0 (-0.08 to 0.08) | 20.05 (13.62 to 28.43) | 19.24 (13.01 to 28.1) | -0.15 (-0.2 to -0.09) |
| Mauritania | 461 (331 to 634) | 758 (529 to 1061) | 0.65 (0.49 to 0.82) | 22.42 (16.09 to 30.87) | 17.25 (12.04 to 24.14) | -0.75 (-0.87 to -0.63) | 21.74 (15.44 to 30.36) | 17.46 (12.08 to 25.06) | -0.65 (-0.74 to -0.55) |
| Mauritius | 213 (146 to 307) | 228 (151 to 342) | 0.07 (-0.06 to 0.21) | 19.47 (13.33 to 27.97) | 17.93 (11.91 to 26.87) | -0.04 (-0.14 to 0.06) | 18.73 (12.86 to 26.98) | 17.82 (11.83 to 26.57) | 0.06 (-0.05 to 0.17) |
| Mexico | 52580 (32983 to 78596) | 48577 (31039 to 73639) | -0.08 (-0.12 to -0.02) | 61.58 (38.63 to 92.04) | 37.58 (24.01 to 56.96) | -0.5 (-0.93 to -0.06) | 58.76 (37.34 to 88.81) | 37.8 (24.21 to 57.38) | -0.39 (-0.8 to 0.03) |
| Micronesia (Federated States of) | 21 (14 to 29) | 21 (14 to 31) | 0.04 (-0.05 to 0.14) | 19.88 (13.59 to 28.32) | 20.89 (14.06 to 30.31) | 0.08 (-0.65 to 0.81) | 20.79 (14.31 to 29.68) | 21.11 (14.14 to 30.81) | 0 (-0.7 to 0.69) |
| Monaco | 8 (5 to 12) | 10 (6 to 15) | 0.2 (0.14 to 0.25) | 27.17 (17.63 to 39.46) | 26.14 (16.47 to 38.86) | -0.1 (-0.18 to -0.02) | 27.07 (17.37 to 39.27) | 25.72 (16.16 to 38.28) | -0.12 (-0.21 to -0.03) |
| Mongolia | 1062 (724 to 1526) | 1708 (1115 to 2540) | 0.61 (0.46 to 0.79) | 49.2 (33.56 to 70.73) | 51.2 (33.43 to 76.14) | 0.23 (0.06 to 0.41) | 46.09 (31.34 to 66.75) | 50.1 (32.61 to 74.62) | 0.35 (0.19 to 0.51) |
| Montenegro | 481 (305 to 741) | 382 (239 to 603) | -0.21 (-0.24 to -0.17) | 76.83 (48.67 to 118.37) | 61.87 (38.62 to 97.64) | -0.7 (-0.76 to -0.63) | 76.63 (48.84 to 117.78) | 64.95 (40.88 to 101.05) | -0.53 (-0.6 to -0.46) |
| Morocco | 10250 (7068 to 14410) | 11896 (8004 to 17293) | 0.16 (0.05 to 0.31) | 40.42 (27.87 to 56.83) | 32 (21.53 to 46.52) | -0.87 (-0.97 to -0.77) | 38.92 (26.73 to 54.4) | 31.89 (21.38 to 46.54) | -0.73 (-0.82 to -0.65) |
| Mozambique | 5148 (3138 to 9049) | 6818 (4800 to 9284) | 0.32 (-0.1 to 0.75) | 38.53 (23.49 to 67.73) | 21.94 (15.45 to 29.88) | -0.76 (-1.41 to -0.11) | 34.58 (21.49 to 59.72) | 21.36 (15.03 to 29.15) | -0.5 (-1.1 to 0.11) |
| Myanmar | 13451 (9364 to 18691) | 25794 (17871 to 37867) | 0.92 (0.5 to 1.65) | 33.26 (23.16 to 46.22) | 45.72 (31.67 to 67.12) | 0.02 (-1.44 to 1.5) | 32.2 (22.41 to 45.11) | 45.01 (31.19 to 65.81) | 0.16 (-1.31 to 1.65) |
| Namibia | 284 (204 to 389) | 483 (343 to 669) | 0.7 (0.59 to 0.82) | 20.19 (14.56 to 27.71) | 19.85 (14.12 to 27.52) | -0.24 (-0.53 to 0.05) | 19.88 (14.33 to 27.32) | 19.53 (13.86 to 27.33) | -0.26 (-0.52 to 0) |
| Nauru | 2 (2 to 3) | 3 (2 to 4) | 0.16 (0.08 to 0.25) | 21.98 (14.94 to 31.45) | 23.67 (15.96 to 34.24) | 0.13 (0.01 to 0.25) | 22.66 (15.47 to 32.55) | 24.25 (16.14 to 35.56) | 0.12 (0.02 to 0.22) |
| Nepal | 5759 (3759 to 8815) | 9940 (6061 to 16287) | 0.73 (0.54 to 0.91) | 29.58 (19.31 to 45.28) | 31.93 (19.47 to 52.32) | -0.11 (-1.33 to 1.13) | 30.26 (19.49 to 46.73) | 31.92 (19.74 to 52.83) | -0.09 (-1.2 to 1.04) |
| Netherlands | 4038 (2638 to 5796) | 5223 (3137 to 8304) | 0.29 (0.11 to 0.51) | 27.06 (17.68 to 38.84) | 30.34 (18.23 to 48.25) | 0.57 (0.21 to 0.94) | 26.51 (17.36 to 37.71) | 25.67 (15.96 to 38.75) | 0.06 (-0.21 to 0.34) |
| New Zealand | 2249 (1434 to 3303) | 2869 (1814 to 4209) | 0.28 (0.18 to 0.37) | 65.8 (41.95 to 96.64) | 55.5 (35.08 to 81.42) | -0.44 (-0.5 to -0.38) | 65.9 (41.7 to 96.2) | 57 (35.85 to 83.58) | -0.39 (-0.47 to -0.3) |
| Nicaragua | 1634 (1134 to 2394) | 1989 (1322 to 3000) | 0.22 (0.09 to 0.36) | 42.03 (29.17 to 61.59) | 29.83 (19.82 to 44.98) | -1.14 (-1.73 to -0.56) | 36.07 (24.45 to 52.31) | 30.07 (19.9 to 46.01) | -0.61 (-1.19 to -0.04) |
| Niger | 2046 (1479 to 2775) | 6246 (4390 to 8586) | 2.05 (1.81 to 2.43) | 25.47 (18.41 to 34.54) | 24.95 (17.53 to 34.29) | 0.11 (-0.2 to 0.42) | 23.02 (16.55 to 31.3) | 23.32 (16.71 to 31.52) | 0.25 (-0.02 to 0.52) |
| Nigeria | 18574 (13228 to 25958) | 44214 (31526 to 60116) | 1.38 (1.16 to 1.73) | 20.63 (14.69 to 28.83) | 19.13 (13.64 to 26.01) | -0.17 (-0.41 to 0.07) | 19.55 (13.7 to 27.52) | 18.86 (13.28 to 25.94) | -0.04 (-0.25 to 0.17) |
| Niue | 0 (0 to 1) | 0 (0 to 0) | -0.29 (-0.36 to -0.22) | 19.4 (13.07 to 28.11) | 18.94 (12.52 to 28.53) | -0.26 (-0.98 to 0.46) | 19.77 (13.29 to 28.56) | 19 (12.53 to 28.63) | -0.31 (-1.04 to 0.43) |
| North Macedonia | 1328 (876 to 1951) | 1198 (768 to 1857) | -0.1 (-0.23 to 0.06) | 66.63 (43.96 to 97.94) | 55.04 (35.29 to 85.34) | -0.71 (-0.86 to -0.56) | 66.35 (43.84 to 97.07) | 59.14 (37.72 to 92.12) | -0.46 (-0.6 to -0.31) |
| Northern Mariana Islands | 13 (9 to 19) | 13 (8 to 19) | -0.04 (-0.16 to 0.08) | 29.09 (19.44 to 41.55) | 26.01 (17.28 to 38.56) | -0.53 (-0.73 to -0.33) | 28.35 (18.54 to 40.85) | 26.56 (17.53 to 39.76) | -0.23 (-0.41 to -0.05) |
| Norway | 1651 (1061 to 2471) | 1690 (1016 to 2691) | 0.02 (-0.08 to 0.12) | 38.88 (24.99 to 58.19) | 31.2 (18.75 to 49.67) | -0.71 (-0.82 to -0.59) | 36.37 (23.23 to 52.31) | 27.77 (17.19 to 42.19) | -0.81 (-0.95 to -0.67) |
| Oman | 1059 (695 to 1539) | 1840 (1218 to 2661) | 0.74 (0.49 to 1.04) | 53.34 (35.04 to 77.56) | 39.11 (25.9 to 56.58) | -0.93 (-1.09 to -0.76) | 52.94 (34.99 to 78.1) | 39.76 (26.49 to 58.2) | -1.05 (-1.17 to -0.93) |
| Pakistan | 20680 (14436 to 28943) | 41087 (28924 to 56785) | 0.99 (0.87 to 1.15) | 18.61 (12.99 to 26.04) | 17.44 (12.28 to 24.11) | 0.59 (-0.33 to 1.53) | 18.46 (12.69 to 25.79) | 17.27 (12.1 to 23.73) | 0.46 (-0.37 to 1.29) |
| Palau | 5 (3 to 7) | 6 (4 to 9) | 0.27 (0.14 to 0.41) | 31.29 (20.56 to 45.96) | 33.44 (21.31 to 51.13) | 0.18 (0.14 to 0.22) | 31.32 (20.58 to 46.62) | 33.53 (21.46 to 51.39) | 0.23 (0.2 to 0.25) |
| Palestine | 1327 (826 to 2292) | 1950 (1316 to 2757) | 0.47 (0.1 to 0.91) | 64.85 (40.38 to 111.98) | 37.96 (25.63 to 53.69) | -1.74 (-4.21 to 0.8) | 57.62 (36.83 to 97.94) | 35.49 (23.97 to 49.8) | -1.61 (-3.98 to 0.82) |
| Panama | 933 (623 to 1378) | 1312 (854 to 1956) | 0.41 (0.33 to 0.48) | 39.04 (26.06 to 57.68) | 30.58 (19.89 to 45.58) | -0.89 (-0.95 to -0.84) | 36.67 (24.39 to 54.24) | 30.88 (20.12 to 46.15) | -0.65 (-0.71 to -0.6) |
| Papua New Guinea | 869 (592 to 1242) | 2603 (1776 to 3706) | 1.99 (1.75 to 2.25) | 21.19 (14.42 to 30.26) | 24.88 (16.98 to 35.42) | -0.41 (-1.35 to 0.54) | 21.89 (14.88 to 31.83) | 26.26 (17.86 to 38.4) | -0.22 (-1.08 to 0.64) |
| Paraguay | 1521 (992 to 2319) | 2543 (1715 to 3671) | 0.67 (0.56 to 0.82) | 37.61 (24.54 to 57.36) | 35.47 (23.93 to 51.21) | -0.24 (-0.33 to -0.15) | 36.25 (23.66 to 54.94) | 34.58 (23.29 to 50.16) | -0.21 (-0.29 to -0.13) |
| Peru | 11838 (8066 to 18445) | 10397 (7038 to 15290) | -0.12 (-0.48 to 0.24) | 54.72 (37.28 to 85.26) | 28.66 (19.4 to 42.16) | -1.41 (-1.85 to -0.97) | 49.01 (34.39 to 75.01) | 28.36 (19.25 to 41.62) | -1.16 (-1.55 to -0.77) |
| Philippines | 22445 (15871 to 30957) | 22967 (15565 to 32096) | 0.02 (-0.2 to 0.22) | 35.62 (25.19 to 49.13) | 20.28 (13.74 to 28.34) | -0.77 (-1.32 to -0.22) | 33.93 (24.15 to 46.76) | 20.16 (13.72 to 28.46) | -0.73 (-1.23 to -0.22) |
| Poland | 31447 (20523 to 47525) | 24057 (14918 to 38306) | -0.23 (-0.32 to -0.15) | 82.38 (53.76 to 124.5) | 62.91 (39.01 to 100.18) | -0.96 (-1.06 to -0.86) | 82.63 (54.15 to 124.57) | 62.92 (39.28 to 97.89) | -0.95 (-1.03 to -0.86) |
| Portugal | 3717 (2501 to 5241) | 2560 (1672 to 3730) | -0.31 (-0.4 to -0.22) | 36.67 (24.67 to 51.7) | 24.14 (15.76 to 35.16) | -1.58 (-1.68 to -1.48) | 37 (24.87 to 52.01) | 22.65 (14.79 to 32.65) | -1.79 (-1.87 to -1.72) |
| Puerto Rico | 1115 (748 to 1646) | 1101 (716 to 1693) | -0.01 (-0.15 to 0.15) | 30.88 (20.71 to 45.57) | 33.43 (21.74 to 51.39) | 0.4 (0.12 to 0.68) | 30.83 (20.7 to 45.43) | 33.87 (22.23 to 49.84) | 0.47 (0.17 to 0.76) |
| Qatar | 218 (145 to 311) | 1253 (815 to 1862) | 4.75 (4.09 to 5.56) | 48.98 (32.68 to 69.9) | 42.08 (27.37 to 62.56) | -0.08 (-0.27 to 0.1) | 43.22 (28.92 to 62.05) | 37.37 (24.51 to 56.07) | -0.38 (-0.5 to -0.26) |
| Republic of Korea | 21162 (13939 to 29305) | 16011 (10203 to 23898) | -0.24 (-0.35 to -0.11) | 47.83 (31.5 to 66.23) | 31.05 (19.78 to 46.34) | -1.67 (-1.76 to -1.57) | 47.31 (31.3 to 65.73) | 28.87 (18.39 to 42.33) | -1.84 (-1.93 to -1.76) |
| Republic of Moldova | 3258 (2127 to 4785) | 1585 (1041 to 2386) | -0.51 (-0.55 to -0.48) | 73.26 (47.84 to 107.6) | 44.09 (28.97 to 66.41) | -1.94 (-2.09 to -1.78) | 73.09 (47.77 to 107.42) | 46.54 (30.46 to 69.87) | -1.78 (-1.94 to -1.62) |
| Romania | 20763 (13153 to 31615) | 11877 (7374 to 18452) | -0.43 (-0.46 to -0.39) | 88.81 (56.26 to 135.22) | 62.71 (38.93 to 97.43) | -1.31 (-1.37 to -1.24) | 91 (57.79 to 137.3) | 67.8 (42.83 to 104.39) | -1.1 (-1.16 to -1.05) |
| Russian Federation | 127097 (82072 to 187430) | 91905 (58992 to 138832) | -0.28 (-0.35 to -0.2) | 84.18 (54.36 to 124.14) | 63.45 (40.73 to 95.84) | -1.22 (-1.63 to -0.81) | 85.45 (55.7 to 125.25) | 65.26 (42.49 to 98.55) | -1.2 (-1.55 to -0.85) |
| Rwanda | 4941 (2805 to 9338) | 2153 (1472 to 3044) | -0.56 (-0.79 to -0.24) | 68.73 (39.01 to 129.89) | 16.22 (11.09 to 22.94) | -5.76 (-8.14 to -3.32) | 60.31 (35.3 to 111.92) | 16.47 (11.36 to 23.51) | -5.35 (-7.72 to -2.91) |
| Saint Kitts and Nevis | 13 (9 to 20) | 18 (12 to 28) | 0.38 (0.24 to 0.52) | 31.63 (21.53 to 47.39) | 30.76 (20.15 to 47.14) | -0.04 (-0.24 to 0.16) | 31.22 (21.31 to 46.84) | 32.23 (21.38 to 49.24) | 0.18 (0.01 to 0.35) |
| Saint Lucia | 37 (26 to 53) | 45 (30 to 67) | 0.21 (0.09 to 0.34) | 27.35 (18.96 to 39.06) | 25.42 (16.68 to 37.6) | -0.3 (-0.47 to -0.14) | 26.28 (18.23 to 37.39) | 26.71 (17.81 to 39.88) | 0.03 (-0.14 to 0.19) |
| Saint Vincent and the Grenadines | 30 (21 to 43) | 31 (21 to 47) | 0.04 (-0.06 to 0.15) | 27.53 (19.05 to 39.69) | 27.52 (18.14 to 41.42) | -0.14 (-0.34 to 0.07) | 26.1 (18.05 to 38.11) | 28.04 (18.58 to 41.71) | 0.12 (-0.08 to 0.33) |
| Samoa | 40 (28 to 55) | 39 (26 to 57) | -0.01 (-0.28 to 0.2) | 23.6 (16.68 to 32.59) | 18.39 (12.35 to 26.87) | -0.13 (-1.39 to 1.15) | 23.31 (16.32 to 31.56) | 19.29 (12.85 to 28.33) | -0.02 (-1.2 to 1.19) |
| San Marino | 7 (4 to 10) | 9 (6 to 14) | 0.37 (0.25 to 0.48) | 29.08 (18.83 to 41.44) | 28.8 (18.29 to 42.44) | 0.03 (-0.03 to 0.1) | 28.9 (18.42 to 41.78) | 27.27 (17.32 to 40.42) | -0.12 (-0.2 to -0.05) |
| Sao Tome and Principe | 29 (20 to 41) | 58 (40 to 82) | 1.02 (0.76 to 1.46) | 23.53 (16.23 to 33.88) | 26.63 (18.46 to 37.99) | -0.04 (-0.18 to 0.1) | 21.41 (14.65 to 31.31) | 27.38 (18.77 to 39.57) | 0.39 (0.27 to 0.5) |
| Saudi Arabia | 10562 (6736 to 16029) | 32300 (20100 to 50052) | 2.06 (1.63 to 2.44) | 66.61 (42.48 to 101.09) | 85.67 (53.31 to 132.75) | 1.09 (0.92 to 1.27) | 67.55 (43.01 to 101.38) | 74.93 (46.9 to 114.87) | 0.51 (0.4 to 0.61) |
| Senegal | 1586 (1131 to 2208) | 2613 (1801 to 3709) | 0.65 (0.45 to 0.85) | 20.78 (14.82 to 28.93) | 16.48 (11.35 to 23.38) | -0.93 (-1.22 to -0.64) | 19.01 (13.41 to 26.41) | 16.73 (11.53 to 24.12) | -0.59 (-0.84 to -0.33) |
| Serbia | 6364 (4193 to 9609) | 4916 (3051 to 7644) | -0.23 (-0.3 to -0.14) | 66.09 (43.54 to 99.79) | 55.12 (34.21 to 85.7) | -1.22 (-1.95 to -0.48) | 68.61 (45.24 to 103.82) | 58.33 (36.64 to 90.88) | -1.14 (-1.89 to -0.39) |
| Seychelles | 16 (11 to 24) | 21 (14 to 30) | 0.25 (0.11 to 0.38) | 22.58 (15.24 to 33.13) | 19.53 (12.96 to 28.61) | -0.55 (-0.74 to -0.35) | 22.44 (15.09 to 32.89) | 19.35 (12.81 to 28.37) | -0.55 (-0.76 to -0.34) |
| Sierra Leone | 852 (608 to 1177) | 1520 (1069 to 2119) | 0.78 (0.67 to 0.91) | 20.52 (14.65 to 28.35) | 17.14 (12.05 to 23.89) | -4.63 (-6.46 to -2.76) | 18.26 (12.79 to 25.55) | 16.92 (11.76 to 24.23) | -4.32 (-6.14 to -2.47) |
| Singapore | 997 (655 to 1433) | 1377 (886 to 2014) | 0.38 (0.27 to 0.5) | 32.72 (21.5 to 47.03) | 24.05 (15.47 to 35.16) | -1.19 (-1.3 to -1.09) | 31.4 (20.76 to 44.99) | 25.48 (16.31 to 37.54) | -0.86 (-0.95 to -0.77) |
| Slovakia | 4919 (3093 to 7551) | 4120 (2505 to 6587) | -0.16 (-0.23 to -0.1) | 93.11 (58.54 to 142.92) | 75.89 (46.14 to 121.32) | -0.67 (-0.7 to -0.64) | 92.47 (58.59 to 140.87) | 76.1 (47.02 to 119.59) | -0.62 (-0.65 to -0.58) |
| Slovenia | 2228 (1394 to 3439) | 1869 (1126 to 3051) | -0.16 (-0.26 to -0.05) | 112.89 (70.61 to 174.27) | 90.29 (54.41 to 147.39) | -0.34 (-0.57 to -0.11) | 111.46 (69.96 to 171.92) | 84.43 (51.6 to 131.97) | -0.51 (-0.78 to -0.23) |
| Solomon Islands | 82 (54 to 121) | 201 (129 to 310) | 1.46 (1.21 to 1.72) | 24.12 (15.91 to 35.74) | 29.39 (18.86 to 45.33) | 0.77 (0.48 to 1.06) | 27.93 (18.34 to 41.89) | 32.55 (20.54 to 51.32) | 0.62 (0.39 to 0.85) |
| Somalia | 5075 (2856 to 9635) | 7253 (4939 to 11081) | 0.43 (0.08 to 0.87) | 63.92 (35.97 to 121.36) | 33.57 (22.86 to 51.29) | 0.7 (-0.83 to 2.26) | 57.12 (33.21 to 108.06) | 30.99 (21.42 to 46.38) | 0.58 (-0.85 to 2.03) |
| South Africa | 12581 (8988 to 17848) | 12682 (9046 to 17859) | 0.01 (-0.06 to 0.07) | 33.99 (24.28 to 48.22) | 22.31 (15.91 to 31.41) | -1.52 (-1.63 to -1.4) | 32.63 (23.05 to 45.96) | 21.55 (15.44 to 30.43) | -1.54 (-1.65 to -1.42) |
| South Sudan | 1170 (832 to 1649) | 2842 (2021 to 4124) | 1.43 (0.88 to 2.62) | 19.9 (14.16 to 28.05) | 29.38 (20.89 to 42.64) | 1.54 (-0.37 to 3.49) | 18.64 (13.26 to 26.18) | 26.66 (19.34 to 37.95) | 1.44 (-0.34 to 3.25) |
| Spain | 12885 (8623 to 18211) | 12108 (7635 to 18477) | -0.06 (-0.2 to 0.09) | 33.22 (22.23 to 46.96) | 26.58 (16.76 to 40.56) | -0.79 (-0.84 to -0.73) | 33.08 (22.21 to 46.26) | 26.12 (16.48 to 39.46) | -0.79 (-0.86 to -0.71) |
| Sri Lanka | 19207 (10491 to 36953) | 6552 (4239 to 10178) | -0.66 (-0.85 to -0.34) | 112.12 (61.24 to 215.71) | 29.42 (19.03 to 45.7) | -3.72 (-5.33 to -2.09) | 101.59 (56.76 to 193.32) | 28.74 (18.74 to 44.57) | -3.42 (-5.05 to -1.76) |
| Sudan | 14007 (8863 to 24913) | 13001 (8866 to 18376) | -0.07 (-0.46 to 0.38) | 69.96 (44.27 to 124.43) | 29.94 (20.42 to 42.32) | -1.95 (-2.7 to -1.2) | 63.18 (40.59 to 109.67) | 28.62 (19.52 to 40.63) | -1.82 (-2.53 to -1.12) |
| Suriname | 97 (68 to 138) | 143 (97 to 209) | 0.47 (0.38 to 0.59) | 25.16 (17.67 to 35.7) | 24.72 (16.77 to 36.02) | 0 (-0.08 to 0.09) | 23.93 (16.84 to 34.04) | 25.15 (17.14 to 36.61) | 0.22 (0.14 to 0.31) |
| Sweden | 2993 (1890 to 4473) | 3155 (1942 to 4948) | 0.05 (-0.03 to 0.13) | 34.85 (22.01 to 52.08) | 30.41 (18.72 to 47.7) | -0.42 (-0.5 to -0.34) | 33.35 (20.98 to 49.18) | 27.08 (16.93 to 40.48) | -0.65 (-0.73 to -0.57) |
| Switzerland | 3831 (2397 to 5696) | 3286 (1959 to 5252) | -0.14 (-0.26 to -0.04) | 55.79 (34.91 to 82.96) | 36.83 (21.95 to 58.86) | -1.5 (-1.62 to -1.38) | 50.84 (31.82 to 75.82) | 33.47 (20.36 to 51.92) | -1.55 (-1.62 to -1.48) |
| Syrian Arab Republic | 3823 (2579 to 5459) | 5823 (3797 to 8831) | 0.52 (0.18 to 1.32) | 30.06 (20.28 to 42.93) | 41.5 (27.06 to 62.94) | 7.56 (4.86 to 10.33) | 27.81 (18.64 to 39.7) | 40.8 (26.66 to 61.61) | 7.84 (5.1 to 10.66) |
| Taiwan (Province of China) | 6090 (4078 to 8908) | 4216 (2695 to 6455) | -0.31 (-0.39 to -0.23) | 29.87 (20 to 43.69) | 17.84 (11.4 to 27.31) | -2.22 (-2.55 to -1.88) | 29.56 (19.81 to 43.21) | 15.83 (10.48 to 23.49) | -2.56 (-2.94 to -2.18) |
| Tajikistan | 2414 (1635 to 3456) | 3256 (2181 to 4734) | 0.35 (0.28 to 0.42) | 44.96 (30.45 to 64.37) | 32.04 (21.47 to 46.6) | -3.5 (-4.94 to -2.03) | 41.61 (27.89 to 60.56) | 30.51 (20.5 to 44.57) | -3.32 (-4.73 to -1.89) |
| Thailand | 18212 (12425 to 25977) | 18136 (12118 to 26632) | 0 (-0.18 to 0.21) | 32.08 (21.89 to 45.76) | 27.2 (18.17 to 39.94) | -0.72 (-0.94 to -0.51) | 30.79 (20.84 to 43.8) | 26.53 (17.73 to 38.82) | -0.62 (-0.88 to -0.36) |
| Timor-Leste | 688 (367 to 1337) | 285 (201 to 389) | -0.59 (-0.81 to -0.22) | 88.11 (47.04 to 171.18) | 20.37 (14.39 to 27.86) | -7.43 (-9.43 to -5.39) | 79.09 (43.25 to 151.82) | 20.38 (14.28 to 28.31) | -6.94 (-8.86 to -4.97) |
| Togo | 752 (535 to 1044) | 1478 (1036 to 2065) | 0.96 (0.84 to 1.11) | 20.63 (14.68 to 28.63) | 17.65 (12.37 to 24.67) | -0.64 (-0.99 to -0.28) | 19.51 (13.5 to 27.31) | 18.1 (12.61 to 25.74) | -0.37 (-0.68 to -0.06) |
| Tokelau | 0 (0 to 0) | 0 (0 to 0) | -0.15 (-0.25 to -0.04) | 17.7 (11.85 to 25.22) | 17.44 (11.42 to 26.42) | -0.06 (-0.13 to 0.01) | 18.17 (12.15 to 26.02) | 17.39 (11.39 to 26.11) | -0.17 (-0.24 to -0.11) |
| Tonga | 17 (12 to 24) | 15 (10 to 22) | -0.12 (-0.23 to -0.02) | 17.25 (12.03 to 24.2) | 14.17 (9.39 to 20.72) | -0.4 (-0.87 to 0.07) | 17.66 (12.16 to 24.82) | 14.56 (9.63 to 21.41) | -0.42 (-0.83 to -0.01) |
| Trinidad and Tobago | 361 (258 to 502) | 383 (275 to 541) | 0.06 (-0.02 to 0.17) | 29.93 (21.4 to 41.64) | 27.51 (19.71 to 38.83) | 0.3 (0 to 0.59) | 29.02 (20.68 to 40.37) | 29.84 (21.66 to 42.25) | 0.68 (0.36 to 1) |
| Tunisia | 3210 (2147 to 4624) | 3690 (2427 to 5435) | 0.15 (0.03 to 0.29) | 38.45 (25.71 to 55.38) | 31.16 (20.5 to 45.9) | -0.6 (-0.72 to -0.48) | 36.63 (24.72 to 52.71) | 31.53 (20.78 to 46.36) | -0.42 (-0.53 to -0.31) |
| Turkmenistan | 1603 (1079 to 2319) | 1703 (1140 to 2537) | 0.06 (-0.04 to 0.18) | 43.32 (29.17 to 62.7) | 33.02 (22.11 to 49.17) | -1.21 (-1.54 to -0.88) | 38.7 (26.23 to 56.24) | 32.1 (21.45 to 47.83) | -0.96 (-1.31 to -0.61) |
| Tuvalu | 2 (1 to 3) | 2 (2 to 4) | 0.26 (0.13 to 0.41) | 20.18 (13.88 to 28.6) | 19.63 (13.1 to 28.48) | -0.65 (-0.88 to -0.42) | 20.54 (14.05 to 29.24) | 19.77 (13.26 to 28.7) | -0.71 (-0.96 to -0.47) |
| Uganda | 4448 (3155 to 6332) | 7610 (5419 to 10364) | 0.71 (0.24 to 1.07) | 25.73 (18.24 to 36.62) | 17.57 (12.51 to 23.93) | -1.36 (-1.98 to -0.73) | 23.05 (16.38 to 32.07) | 17.19 (12.04 to 24.05) | -1.09 (-1.64 to -0.53) |
| Ukraine | 42543 (27356 to 63238) | 27988 (18246 to 41955) | -0.34 (-0.38 to -0.31) | 80.71 (51.9 to 119.97) | 64.97 (42.36 to 97.39) | -1.05 (-1.26 to -0.83) | 83.12 (53.58 to 123.42) | 68.79 (45.07 to 102.76) | -0.91 (-1.14 to -0.69) |
| United Arab Emirates | 852 (568 to 1234) | 4177 (2767 to 6090) | 3.9 (3.43 to 4.43) | 45.53 (30.34 to 65.96) | 43.37 (28.73 to 63.23) | 0.2 (0.04 to 0.37) | 42.28 (28.28 to 61.18) | 38.55 (25.71 to 56.95) | -0.26 (-0.3 to -0.23) |
| United Kingdom | 17432 (11235 to 24704) | 17571 (10765 to 27356) | 0.01 (-0.1 to 0.12) | 30.42 (19.61 to 43.11) | 25.9 (15.87 to 40.32) | -0.44 (-0.53 to -0.35) | 29.82 (19.22 to 42.59) | 24.38 (15.26 to 36.52) | -0.59 (-0.71 to -0.48) |
| United Republic of Tanzania | 5289 (3769 to 7256) | 10163 (6983 to 14340) | 0.92 (0.8 to 1.04) | 20.47 (14.59 to 28.08) | 17.39 (11.95 to 24.54) | -0.47 (-0.53 to -0.41) | 18.83 (13.15 to 26.13) | 17.14 (11.71 to 24.65) | -0.29 (-0.33 to -0.24) |
| United States of America | 82320 (54789 to 112967) | 100249 (65951 to 144601) | 0.22 (0.06 to 0.39) | 32.4 (21.56 to 44.46) | 30.14 (19.83 to 43.47) | -0.21 (-0.34 to -0.08) | 31.78 (21.02 to 43.64) | 26.53 (17.88 to 37.02) | -0.62 (-0.77 to -0.46) |
| United States Virgin Islands | 30 (21 to 43) | 22 (15 to 33) | -0.26 (-0.35 to -0.15) | 28.36 (19.58 to 40.9) | 25.87 (17.22 to 38.49) | -0.5 (-0.64 to -0.36) | 28.44 (19.64 to 41.12) | 26.27 (17.74 to 39.1) | -0.47 (-0.67 to -0.27) |
| Uruguay | 1384 (848 to 2219) | 1217 (758 to 1893) | -0.12 (-0.2 to -0.01) | 44.09 (27 to 70.67) | 35.73 (22.27 to 55.6) | -0.83 (-0.88 to -0.77) | 44.8 (27.32 to 71.83) | 36.7 (22.83 to 56.44) | -0.79 (-0.84 to -0.73) |
| Uzbekistan | 9171 (6172 to 13283) | 11635 (7869 to 16837) | 0.27 (0.21 to 0.33) | 43.76 (29.45 to 63.38) | 33.99 (22.99 to 49.18) | -0.91 (-1.06 to -0.76) | 40.37 (27.49 to 58.45) | 33.44 (22.52 to 48.45) | -0.71 (-0.84 to -0.59) |
| Vanuatu | 26 (18 to 37) | 53 (36 to 76) | 1.02 (0.9 to 1.13) | 17.31 (11.69 to 24.49) | 16.98 (11.43 to 24.22) | -0.27 (-0.88 to 0.35) | 17.85 (12.04 to 25.28) | 17.44 (11.79 to 24.93) | -0.25 (-0.77 to 0.28) |
| Venezuela (Bolivarian Republic of) | 9672 (6350 to 14246) | 10376 (6722 to 15577) | 0.07 (0.01 to 0.15) | 51.43 (33.76 to 75.75) | 38.97 (25.24 to 58.5) | -1.14 (-1.68 to -0.59) | 47.1 (30.95 to 69.8) | 41.03 (26.54 to 61.26) | -0.72 (-1.29 to -0.16) |
| Viet Nam | 17047 (11679 to 24779) | 27730 (18128 to 41337) | 0.63 (0.38 to 0.92) | 24.99 (17.12 to 36.32) | 27.66 (18.08 to 41.23) | 0.42 (0.31 to 0.54) | 26.1 (17.88 to 37.55) | 28.07 (18.32 to 42.22) | 0.3 (0.18 to 0.42) |
| Yemen | 4910 (3305 to 7003) | 35253 (19523 to 69397) | 6.18 (3.05 to 14.39) | 36.01 (24.24 to 51.36) | 104.78 (58.03 to 206.27) | 4.07 (2.77 to 5.38) | 34.01 (23.11 to 48.16) | 93.92 (53.04 to 182.95) | 3.86 (2.61 to 5.12) |
| Zambia | 1613 (1152 to 2237) | 3335 (2318 to 4758) | 1.07 (0.9 to 1.25) | 20.33 (14.52 to 28.18) | 17.09 (11.88 to 24.38) | -0.53 (-0.63 to -0.42) | 19.02 (13.31 to 26.31) | 17.93 (12.37 to 25.7) | -0.17 (-0.25 to -0.08) |
| Zimbabwe | 1928 (1356 to 2629) | 2682 (1889 to 3741) | 0.39 (0.33 to 0.46) | 18.64 (13.11 to 25.42) | 17.2 (12.11 to 23.99) | -0.21 (-0.4 to -0.03) | 18.95 (13.53 to 26.46) | 17.61 (12.42 to 24.64) | -0.22 (-0.37 to -0.07) |

CR, crude rate; ASR, age-standardized rate; EAPC, estimated annual percentage change; UI, uncertainty interval; CI, confidence interval.
