# Supplementary material for: A comprehensive study of hip dislocation: global health burden from 1990 to 2021 and its predictions to 2030
Source: Front Public Health. 2025 Sep 9;13:1594523. doi: 10.3389/fpubh.2025.1594523 (PMC12454331; doi:10.3389/fpubh.2025.1594523)
Supplement: Supplementary file 4 [file Table_2.docx]

Table S2. YLDs of hip dislocation in 1990 and 2021 for both sexes in countries and territories, with EAPC from 1990 to 2021.

| **Location** | **Number in 1990 (95% UI)** | **Number in 2021 (95% UI)** | **Number change rate (95% UI)** | **CR in 1990 (95% UI)** | **CR in 2021 (95% UI)** | **EAPC of CR, % per year (95% CI)** | **ASR in 1990 (95% UI)** | **ASR in 2021 (95% UI)** | **EAPC of ASR, % per year (95% CI)** |
| --- | --- | --- | --- | --- | --- | --- | --- | --- | --- |
| Afghanistan | 845 (164 to 2932) | 1796 (578 to 4540) | 1.13 (0.34 to 4.08) | 8.49 (1.65 to 29.49) | 5.75 (1.85 to 14.54) | -1.62 (-1.86 to -1.37) | 10.25 (2.04 to 34.92) | 9.03 (2.61 to 24.67) | -0.61 (-0.68 to -0.53) |
| Albania | 56 (28 to 96) | 62 (31 to 107) | 0.11 (-0.01 to 0.26) | 1.7 (0.85 to 2.91) | 2.33 (1.17 to 4) | 0.89 (0.69 to 1.08) | 2.03 (1.03 to 3.49) | 1.83 (0.92 to 3.15) | -0.53 (-0.72 to -0.35) |
| Algeria | 276 (145 to 485) | 506 (257 to 887) | 0.84 (0.54 to 1.32) | 1.09 (0.57 to 1.92) | 1.14 (0.58 to 2.01) | 0.1 (-0.07 to 0.27) | 1.57 (0.84 to 2.72) | 1.19 (0.61 to 2.1) | -0.96 (-1.05 to -0.87) |
| American Samoa | 0 (0 to 0) | 0 (0 to 1) | 0.72 (0.41 to 1.28) | 0.54 (0.29 to 0.96) | 0.91 (0.47 to 1.67) | 2.11 (1.85 to 2.37) | 0.8 (0.42 to 1.42) | 0.92 (0.47 to 1.68) | 0.85 (0.63 to 1.08) |
| Andorra | 1 (1 to 2) | 2 (1 to 4) | 1.33 (1.08 to 1.61) | 1.82 (0.95 to 3.13) | 2.69 (1.41 to 4.74) | 1.19 (1.08 to 1.31) | 1.69 (0.89 to 2.92) | 1.68 (0.88 to 2.95) | -0.03 (-0.09 to 0.03) |
| Angola | 213 (87 to 485) | 397 (166 to 859) | 0.86 (0.66 to 1.19) | 2.08 (0.85 to 4.72) | 1.21 (0.51 to 2.63) | -2.03 (-2.42 to -1.63) | 2.34 (0.99 to 4.99) | 2.06 (0.87 to 4.5) | -0.59 (-0.91 to -0.27) |
| Antigua and Barbuda | 0 (0 to 1) | 1 (0 to 2) | 0.88 (0.63 to 1.19) | 0.77 (0.39 to 1.36) | 0.98 (0.51 to 1.69) | 0.73 (0.59 to 0.87) | 0.83 (0.43 to 1.46) | 0.86 (0.45 to 1.48) | 0.02 (-0.18 to 0.22) |
| Argentina | 444 (232 to 770) | 642 (330 to 1098) | 0.45 (0.31 to 0.61) | 1.34 (0.7 to 2.33) | 1.41 (0.73 to 2.41) | 0.08 (-0.01 to 0.17) | 1.38 (0.72 to 2.39) | 1.24 (0.64 to 2.11) | -0.41 (-0.52 to -0.3) |
| Armenia | 89 (42 to 189) | 64 (34 to 115) | -0.28 (-0.42 to 0.01) | 2.61 (1.24 to 5.52) | 2.13 (1.13 to 3.82) | -1.09 (-1.33 to -0.86) | 2.8 (1.32 to 5.9) | 1.85 (0.95 to 3.42) | -2.01 (-2.32 to -1.69) |
| Australia | 414 (216 to 708) | 694 (368 to 1210) | 0.68 (0.54 to 0.84) | 2.46 (1.28 to 4.2) | 2.69 (1.43 to 4.69) | 0.38 (0.32 to 0.44) | 2.23 (1.16 to 3.84) | 1.86 (0.98 to 3.23) | -0.5 (-0.55 to -0.44) |
| Austria | 186 (97 to 322) | 197 (104 to 350) | 0.06 (-0.04 to 0.17) | 2.4 (1.24 to 4.14) | 2.2 (1.15 to 3.9) | -0.14 (-0.2 to -0.07) | 1.84 (0.95 to 3.18) | 1.35 (0.71 to 2.39) | -0.92 (-0.98 to -0.87) |
| Azerbaijan | 79 (41 to 140) | 131 (68 to 229) | 0.65 (0.32 to 1.26) | 1.08 (0.56 to 1.91) | 1.25 (0.65 to 2.18) | -0.14 (-0.33 to 0.04) | 1.26 (0.66 to 2.18) | 1.17 (0.6 to 2.05) | -0.84 (-1.02 to -0.66) |
| Bahamas | 2 (1 to 3) | 4 (2 to 7) | 1.18 (0.82 to 1.65) | 0.74 (0.39 to 1.29) | 1.06 (0.57 to 1.87) | 1.08 (1.01 to 1.14) | 0.9 (0.47 to 1.57) | 0.99 (0.53 to 1.76) | 0.21 (0.14 to 0.27) |
| Bahrain | 4 (2 to 7) | 13 (7 to 23) | 2.37 (1.84 to 3.09) | 0.77 (0.4 to 1.33) | 0.85 (0.44 to 1.52) | -0.23 (-0.48 to 0.01) | 1 (0.53 to 1.72) | 0.84 (0.43 to 1.46) | -1.22 (-1.49 to -0.95) |
| Bangladesh | 482 (243 to 894) | 1067 (525 to 1983) | 1.21 (0.76 to 1.77) | 0.44 (0.22 to 0.82) | 0.65 (0.32 to 1.2) | 0.05 (-0.22 to 0.31) | 0.61 (0.31 to 1.1) | 0.67 (0.33 to 1.25) | -0.53 (-0.74 to -0.32) |
| Barbados | 2 (1 to 3) | 3 (1 to 5) | 0.5 (0.3 to 0.74) | 0.75 (0.39 to 1.33) | 0.96 (0.5 to 1.63) | 0.57 (0.47 to 0.68) | 0.71 (0.37 to 1.25) | 0.71 (0.37 to 1.23) | -0.18 (-0.31 to -0.05) |
| Belarus | 275 (139 to 480) | 277 (141 to 495) | 0.01 (-0.09 to 0.12) | 2.64 (1.33 to 4.6) | 2.97 (1.52 to 5.31) | 0.26 (-0.08 to 0.59) | 2.32 (1.16 to 4.04) | 2.09 (1.06 to 3.76) | -0.49 (-0.81 to -0.17) |
| Belgium | 237 (120 to 417) | 309 (167 to 529) | 0.3 (0.19 to 0.45) | 2.37 (1.21 to 4.18) | 2.69 (1.45 to 4.62) | 0.6 (0.32 to 0.88) | 1.8 (0.9 to 3.15) | 1.6 (0.86 to 2.75) | -0.19 (-0.5 to 0.13) |
| Belize | 1 (1 to 2) | 4 (2 to 7) | 2.39 (1.86 to 2.97) | 0.66 (0.34 to 1.15) | 0.98 (0.51 to 1.68) | 0.85 (0.67 to 1.03) | 0.92 (0.48 to 1.6) | 1.09 (0.58 to 1.88) | 0.25 (0.04 to 0.46) |
| Benin | 21 (11 to 38) | 55 (29 to 95) | 1.59 (1.19 to 2.09) | 0.44 (0.23 to 0.78) | 0.41 (0.21 to 0.7) | -0.28 (-0.32 to -0.24) | 0.7 (0.36 to 1.22) | 0.66 (0.34 to 1.12) | -0.24 (-0.29 to -0.18) |
| Bermuda | 1 (0 to 1) | 1 (0 to 1) | 0.39 (0.2 to 0.59) | 0.85 (0.45 to 1.45) | 1.1 (0.57 to 1.93) | 0.66 (0.61 to 0.71) | 0.78 (0.41 to 1.35) | 0.7 (0.36 to 1.25) | -0.52 (-0.61 to -0.43) |
| Bhutan | 3 (1 to 5) | 6 (3 to 11) | 1.26 (0.88 to 1.73) | 0.43 (0.22 to 0.77) | 0.81 (0.42 to 1.43) | 1.99 (1.79 to 2.19) | 0.7 (0.36 to 1.22) | 0.89 (0.46 to 1.56) | 0.78 (0.61 to 0.95) |
| Bolivia (Plurinational State of) | 51 (27 to 89) | 96 (50 to 165) | 0.88 (0.63 to 1.18) | 0.8 (0.42 to 1.39) | 0.81 (0.43 to 1.4) | 0.04 (0.01 to 0.07) | 1.11 (0.58 to 1.9) | 0.91 (0.47 to 1.57) | -0.69 (-0.72 to -0.66) |
| Bosnia and Herzegovina | 96 (49 to 167) | 125 (63 to 226) | 0.3 (-0.04 to 0.97) | 2.13 (1.08 to 3.72) | 3.77 (1.92 to 6.84) | 0.98 (0.5 to 1.46) | 2.11 (1.08 to 3.7) | 2.76 (1.41 to 5.06) | -0.03 (-0.57 to 0.51) |
| Botswana | 6 (3 to 11) | 17 (9 to 30) | 1.74 (1.35 to 2.26) | 0.48 (0.25 to 0.86) | 0.73 (0.39 to 1.25) | 1.24 (1.04 to 1.44) | 0.75 (0.39 to 1.33) | 0.84 (0.45 to 1.45) | 0.23 (0 to 0.47) |
| Brazil | 2131 (1122 to 3723) | 3632 (1964 to 6242) | 0.7 (0.58 to 0.9) | 1.43 (0.76 to 2.51) | 1.65 (0.89 to 2.83) | 0.39 (0.34 to 0.45) | 1.83 (0.96 to 3.17) | 1.48 (0.8 to 2.53) | -0.75 (-0.81 to -0.7) |
| Brunei Darussalam | 3 (2 to 5) | 5 (3 to 10) | 0.77 (0.55 to 1.02) | 1.17 (0.6 to 2.04) | 1.19 (0.62 to 2.11) | -0.01 (-0.12 to 0.1) | 1.76 (0.92 to 3) | 1.26 (0.66 to 2.2) | -1.11 (-1.17 to -1.05) |
| Bulgaria | 258 (134 to 455) | 197 (102 to 356) | -0.24 (-0.3 to -0.16) | 2.97 (1.55 to 5.24) | 2.9 (1.5 to 5.25) | -0.11 (-0.14 to -0.08) | 2.45 (1.28 to 4.31) | 1.97 (1.02 to 3.53) | -0.75 (-0.79 to -0.72) |
| Burkina Faso | 45 (24 to 79) | 118 (62 to 212) | 1.61 (1.18 to 2.14) | 0.48 (0.25 to 0.83) | 0.52 (0.27 to 0.93) | 0.1 (-0.05 to 0.26) | 0.75 (0.39 to 1.31) | 0.79 (0.41 to 1.37) | 0.07 (-0.02 to 0.16) |
| Burundi | 24 (13 to 42) | 489 (152 to 1236) | 19.06 (7.41 to 43.06) | 0.44 (0.23 to 0.76) | 3.7 (1.15 to 9.35) | 6.22 (3.82 to 8.68) | 0.69 (0.37 to 1.19) | 5.05 (1.6 to 12.67) | 6.27 (4.42 to 8.15) |
| Cabo Verde | 2 (1 to 3) | 4 (2 to 6) | 0.92 (0.64 to 1.23) | 0.54 (0.29 to 0.93) | 0.66 (0.34 to 1.12) | 0.76 (0.67 to 0.84) | 0.74 (0.39 to 1.31) | 0.71 (0.36 to 1.2) | -0.11 (-0.15 to -0.07) |
| Cambodia | 323 (85 to 1020) | 359 (138 to 924) | 0.11 (-0.1 to 0.75) | 3.15 (0.83 to 9.93) | 2.11 (0.81 to 5.42) | -1.28 (-1.31 to -1.26) | 3.52 (1.07 to 10.4) | 2.43 (0.96 to 6.21) | -1.25 (-1.34 to -1.15) |
| Cameroon | 47 (24 to 82) | 166 (84 to 292) | 2.5 (1.78 to 3.59) | 0.45 (0.23 to 0.79) | 0.52 (0.27 to 0.92) | 0.22 (0.09 to 0.35) | 0.71 (0.36 to 1.21) | 0.77 (0.4 to 1.32) | 0.12 (0.06 to 0.18) |
| Canada | 446 (230 to 793) | 710 (379 to 1201) | 0.59 (0.43 to 0.75) | 1.64 (0.84 to 2.91) | 1.89 (1.01 to 3.21) | 0.59 (0.55 to 0.64) | 1.42 (0.74 to 2.52) | 1.15 (0.6 to 1.91) | -0.63 (-0.67 to -0.6) |
| Central African Republic | 15 (8 to 26) | 67 (31 to 132) | 3.5 (1.94 to 6.8) | 0.55 (0.28 to 0.97) | 1.23 (0.56 to 2.41) | 2.88 (2.44 to 3.32) | 0.84 (0.44 to 1.45) | 1.48 (0.7 to 2.85) | 1.93 (1.65 to 2.21) |
| Chad | 66 (27 to 139) | 130 (61 to 257) | 0.98 (0.67 to 1.51) | 1.09 (0.46 to 2.31) | 0.73 (0.34 to 1.45) | -1.32 (-1.44 to -1.2) | 1.34 (0.58 to 2.72) | 1.32 (0.62 to 2.59) | -0.08 (-0.2 to 0.04) |
| Chile | 156 (83 to 272) | 281 (149 to 492) | 0.8 (0.6 to 1.05) | 1.18 (0.62 to 2.05) | 1.5 (0.79 to 2.62) | 0.89 (0.84 to 0.94) | 1.35 (0.71 to 2.36) | 1.23 (0.64 to 2.13) | -0.24 (-0.29 to -0.18) |
| China | 12079 (6374 to 20796) | 21072 (11095 to 36416) | 0.74 (0.65 to 0.85) | 1.03 (0.54 to 1.77) | 1.48 (0.78 to 2.56) | 0.88 (0.6 to 1.16) | 1.2 (0.63 to 2.05) | 1.11 (0.59 to 1.93) | -0.58 (-0.85 to -0.3) |
| Colombia | 498 (264 to 871) | 740 (393 to 1311) | 0.48 (0.28 to 0.77) | 1.53 (0.81 to 2.68) | 1.51 (0.8 to 2.67) | -0.12 (-0.17 to -0.07) | 1.89 (0.99 to 3.26) | 1.38 (0.73 to 2.45) | -1.11 (-1.16 to -1.05) |
| Comoros | 2 (1 to 4) | 4 (2 to 8) | 1.07 (0.7 to 1.56) | 0.47 (0.24 to 0.81) | 0.6 (0.31 to 1.06) | 0.59 (0.47 to 0.72) | 0.71 (0.37 to 1.22) | 0.71 (0.36 to 1.26) | -0.2 (-0.3 to -0.1) |
| Congo | 12 (6 to 22) | 67 (29 to 142) | 4.42 (2.15 to 9.28) | 0.52 (0.26 to 0.9) | 1.24 (0.53 to 2.63) | 2.72 (1.24 to 4.23) | 0.8 (0.41 to 1.43) | 1.48 (0.66 to 2.99) | 2.01 (1.01 to 3.02) |
| Cook Islands | 0 (0 to 0) | 0 (0 to 0) | 0.37 (0.18 to 0.61) | 0.73 (0.39 to 1.29) | 1.07 (0.56 to 1.89) | 1.3 (1.1 to 1.49) | 0.9 (0.48 to 1.58) | 0.87 (0.45 to 1.54) | -0.08 (-0.26 to 0.09) |
| Costa Rica | 28 (14 to 48) | 56 (30 to 97) | 1.04 (0.8 to 1.33) | 0.91 (0.47 to 1.59) | 1.19 (0.64 to 2.05) | 0.88 (0.85 to 0.92) | 1.2 (0.62 to 2.09) | 1.06 (0.56 to 1.84) | -0.39 (-0.41 to -0.38) |
| Côte d'Ivoire | 50 (25 to 90) | 135 (71 to 231) | 1.68 (1.19 to 2.34) | 0.41 (0.21 to 0.74) | 0.48 (0.26 to 0.83) | 0.55 (0.45 to 0.66) | 0.7 (0.37 to 1.21) | 0.73 (0.39 to 1.24) | 0.09 (0.03 to 0.15) |
| Croatia | 136 (72 to 237) | 157 (82 to 262) | 0.15 (0.01 to 0.4) | 2.8 (1.48 to 4.88) | 3.72 (1.96 to 6.22) | 0.71 (0.56 to 0.87) | 2.43 (1.28 to 4.24) | 2.27 (1.2 to 3.81) | -0.53 (-0.73 to -0.34) |
| Cuba | 125 (66 to 218) | 200 (106 to 338) | 0.6 (0.42 to 0.8) | 1.15 (0.6 to 2.01) | 1.77 (0.94 to 3) | 1.35 (1.32 to 1.38) | 1.17 (0.61 to 2.04) | 1.17 (0.62 to 2.01) | -0.1 (-0.14 to -0.05) |
| Cyprus | 13 (7 to 23) | 24 (12 to 42) | 0.8 (0.64 to 0.99) | 1.71 (0.88 to 2.96) | 1.77 (0.91 to 3.1) | 0.1 (0 to 0.19) | 1.71 (0.89 to 2.96) | 1.33 (0.69 to 2.34) | -0.82 (-0.92 to -0.71) |
| Czechia | 359 (186 to 621) | 317 (166 to 551) | -0.12 (-0.19 to -0.04) | 3.49 (1.81 to 6.03) | 2.98 (1.56 to 5.18) | -0.58 (-0.7 to -0.45) | 2.92 (1.51 to 5.08) | 1.95 (1.01 to 3.43) | -1.38 (-1.51 to -1.25) |
| Democratic People's Republic of Korea | 169 (89 to 294) | 242 (127 to 418) | 0.44 (0.25 to 0.62) | 0.82 (0.43 to 1.43) | 0.92 (0.48 to 1.58) | 0.44 (0.37 to 0.51) | 0.9 (0.48 to 1.57) | 0.76 (0.4 to 1.31) | -0.5 (-0.54 to -0.47) |
| Democratic Republic of the Congo | 195 (101 to 335) | 705 (332 to 1323) | 2.61 (1.47 to 4.99) | 0.51 (0.26 to 0.88) | 0.78 (0.37 to 1.47) | 1.18 (0.57 to 1.79) | 0.79 (0.41 to 1.35) | 1.11 (0.53 to 2.1) | 1.08 (0.7 to 1.46) |
| Denmark | 123 (65 to 218) | 104 (55 to 178) | -0.16 (-0.24 to -0.07) | 2.4 (1.26 to 4.24) | 1.77 (0.93 to 3.03) | -1.04 (-1.13 to -0.95) | 1.71 (0.89 to 3.03) | 1.13 (0.58 to 1.92) | -1.36 (-1.42 to -1.3) |
| Djibouti | 2 (1 to 3) | 8 (4 to 14) | 3 (2.18 to 4.11) | 0.47 (0.25 to 0.82) | 0.62 (0.32 to 1.14) | 0.2 (-0.08 to 0.48) | 0.7 (0.37 to 1.24) | 0.78 (0.41 to 1.41) | -0.12 (-0.33 to 0.09) |
| Dominica | 0 (0 to 1) | 1 (0 to 1) | 0.38 (0.15 to 0.69) | 0.68 (0.36 to 1.16) | 1.02 (0.54 to 1.89) | 1.24 (1.15 to 1.32) | 0.79 (0.41 to 1.32) | 0.9 (0.47 to 1.67) | 0.38 (0.3 to 0.47) |
| Dominican Republic | 44 (23 to 78) | 100 (50 to 180) | 1.26 (0.93 to 1.63) | 0.62 (0.32 to 1.1) | 0.91 (0.46 to 1.63) | 1.34 (1.18 to 1.51) | 0.82 (0.43 to 1.44) | 0.93 (0.47 to 1.67) | 0.46 (0.31 to 0.62) |
| Ecuador | 94 (49 to 175) | 206 (105 to 361) | 1.18 (0.85 to 1.56) | 0.95 (0.49 to 1.75) | 1.14 (0.58 to 2) | 0.64 (0.49 to 0.79) | 1.25 (0.65 to 2.3) | 1.18 (0.6 to 2.07) | -0.2 (-0.36 to -0.04) |
| Egypt | 535 (279 to 924) | 895 (466 to 1522) | 0.67 (0.42 to 0.99) | 0.97 (0.5 to 1.67) | 0.85 (0.44 to 1.44) | -0.45 (-0.53 to -0.36) | 1.29 (0.68 to 2.19) | 1.05 (0.55 to 1.76) | -0.61 (-0.69 to -0.53) |
| El Salvador | 272 (104 to 635) | 198 (90 to 408) | -0.27 (-0.37 to -0.06) | 5.13 (1.96 to 11.97) | 3.08 (1.39 to 6.32) | -1.6 (-1.71 to -1.5) | 5.3 (2.2 to 11.63) | 3.24 (1.45 to 6.74) | -1.64 (-1.69 to -1.58) |
| Equatorial Guinea | 2 (1 to 4) | 6 (3 to 10) | 1.54 (1.1 to 2.08) | 0.53 (0.28 to 0.92) | 0.37 (0.19 to 0.64) | -1.21 (-1.36 to -1.07) | 0.8 (0.42 to 1.38) | 0.61 (0.32 to 1.04) | -0.91 (-0.97 to -0.85) |
| Eritrea | 301 (92 to 774) | 251 (80 to 640) | -0.17 (-0.26 to 0.08) | 8.84 (2.71 to 22.73) | 3.8 (1.21 to 9.7) | -2.86 (-2.99 to -2.73) | 8.83 (2.74 to 21.81) | 5.84 (1.87 to 14.76) | -1.49 (-1.66 to -1.32) |
| Estonia | 53 (27 to 96) | 31 (16 to 55) | -0.41 (-0.48 to -0.35) | 3.4 (1.73 to 6.13) | 2.39 (1.21 to 4.22) | -1.57 (-1.73 to -1.42) | 2.89 (1.47 to 5.19) | 1.6 (0.8 to 2.86) | -2.33 (-2.48 to -2.19) |
| Eswatini | 5 (2 to 8) | 8 (4 to 14) | 0.77 (0.52 to 1.1) | 0.56 (0.29 to 0.98) | 0.69 (0.36 to 1.22) | 0.68 (0.56 to 0.81) | 0.94 (0.49 to 1.64) | 0.91 (0.48 to 1.57) | -0.06 (-0.23 to 0.11) |
| Ethiopia | 531 (261 to 1038) | 846 (379 to 1826) | 0.6 (0.24 to 1.36) | 1.05 (0.52 to 2.05) | 0.78 (0.35 to 1.68) | -1.21 (-1.49 to -0.93) | 1.36 (0.69 to 2.58) | 1.16 (0.53 to 2.46) | -0.66 (-0.87 to -0.45) |
| Fiji | 4 (2 to 7) | 5 (3 to 10) | 0.45 (0.23 to 0.75) | 0.49 (0.26 to 0.87) | 0.59 (0.31 to 1.03) | 0.53 (0.4 to 0.66) | 0.67 (0.35 to 1.17) | 0.63 (0.33 to 1.11) | -0.26 (-0.34 to -0.18) |
| Finland | 145 (75 to 249) | 158 (84 to 270) | 0.09 (0 to 0.19) | 2.89 (1.5 to 4.97) | 2.85 (1.52 to 4.88) | -0.06 (-0.54 to 0.43) | 2.24 (1.16 to 3.88) | 1.63 (0.86 to 2.8) | -1.06 (-1.54 to -0.56) |
| France | 1502 (787 to 2551) | 1753 (921 to 2995) | 0.17 (0.06 to 0.27) | 2.6 (1.36 to 4.42) | 2.64 (1.39 to 4.51) | 0.09 (0.06 to 0.13) | 2.02 (1.05 to 3.48) | 1.53 (0.81 to 2.64) | -0.85 (-0.9 to -0.8) |
| Gabon | 6 (3 to 10) | 10 (5 to 18) | 0.68 (0.44 to 0.95) | 0.58 (0.3 to 1.01) | 0.52 (0.28 to 0.96) | -0.34 (-0.37 to -0.31) | 0.81 (0.42 to 1.39) | 0.7 (0.37 to 1.29) | -0.48 (-0.52 to -0.44) |
| Gambia | 5 (2 to 10) | 10 (5 to 18) | 0.9 (0.41 to 1.51) | 0.54 (0.25 to 1.01) | 0.42 (0.21 to 0.74) | -0.86 (-0.92 to -0.79) | 0.77 (0.38 to 1.4) | 0.7 (0.35 to 1.23) | -0.39 (-0.46 to -0.32) |
| Georgia | 100 (51 to 172) | 87 (45 to 151) | -0.13 (-0.23 to 0.01) | 1.81 (0.93 to 3.12) | 2.42 (1.25 to 4.18) | 1.13 (0.99 to 1.27) | 1.69 (0.86 to 2.9) | 1.88 (0.96 to 3.23) | 0.48 (0.34 to 0.61) |
| Germany | 1648 (855 to 2841) | 1844 (960 to 3082) | 0.12 (0.02 to 0.23) | 2.06 (1.07 to 3.55) | 2.16 (1.12 to 3.61) | 0.19 (0.14 to 0.24) | 1.52 (0.78 to 2.65) | 1.22 (0.63 to 2.06) | -0.74 (-0.77 to -0.71) |
| Ghana | 60 (32 to 104) | 150 (78 to 269) | 1.49 (1.09 to 1.98) | 0.4 (0.21 to 0.7) | 0.44 (0.23 to 0.79) | 0.22 (0.12 to 0.32) | 0.64 (0.33 to 1.1) | 0.63 (0.33 to 1.1) | -0.05 (-0.13 to 0.03) |
| Greece | 214 (114 to 373) | 204 (103 to 351) | -0.05 (-0.14 to 0.07) | 2.06 (1.09 to 3.59) | 2.01 (1.01 to 3.45) | -0.07 (-0.11 to -0.04) | 1.64 (0.85 to 2.87) | 1.18 (0.6 to 2.03) | -1.08 (-1.16 to -1.01) |
| Greenland | 1 (0 to 2) | 1 (1 to 2) | 0.16 (0.03 to 0.3) | 1.61 (0.85 to 2.75) | 1.84 (0.97 to 3.27) | 0.29 (0.21 to 0.36) | 2.09 (1.11 to 3.56) | 1.57 (0.83 to 2.78) | -1.1 (-1.17 to -1.02) |
| Grenada | 1 (1 to 2) | 1 (1 to 2) | 0.2 (0.01 to 0.46) | 1.31 (0.6 to 2.55) | 1.32 (0.67 to 2.41) | 0.19 (0.12 to 0.27) | 1.44 (0.68 to 2.75) | 1.15 (0.59 to 2.06) | -0.71 (-0.78 to -0.64) |
| Guam | 1 (0 to 1) | 1 (1 to 2) | 0.69 (0.46 to 0.99) | 0.6 (0.31 to 1.07) | 0.87 (0.45 to 1.52) | 1.26 (1.22 to 1.29) | 0.75 (0.39 to 1.33) | 0.74 (0.38 to 1.29) | -0.01 (-0.07 to 0.04) |
| Guatemala | 206 (89 to 448) | 290 (141 to 546) | 0.4 (0.13 to 0.82) | 2.46 (1.06 to 5.34) | 1.84 (0.89 to 3.47) | -0.97 (-1.09 to -0.85) | 3.12 (1.38 to 6.41) | 2.25 (1.08 to 4.3) | -1.14 (-1.2 to -1.09) |
| Guinea | 30 (15 to 53) | 69 (36 to 121) | 1.27 (0.85 to 1.8) | 0.5 (0.26 to 0.89) | 0.51 (0.27 to 0.9) | 0.16 (0 to 0.32) | 0.7 (0.36 to 1.22) | 0.77 (0.41 to 1.35) | 0.41 (0.31 to 0.51) |
| Guinea-Bissau | 6 (3 to 9) | 12 (6 to 20) | 1.07 (0.65 to 1.76) | 0.55 (0.28 to 0.94) | 0.56 (0.28 to 0.98) | 0.08 (-0.3 to 0.46) | 0.89 (0.45 to 1.55) | 0.9 (0.46 to 1.52) | 0.07 (-0.15 to 0.3) |
| Guyana | 6 (3 to 11) | 8 (4 to 14) | 0.38 (0.21 to 0.59) | 0.78 (0.39 to 1.35) | 1.09 (0.58 to 1.88) | 1.15 (1.06 to 1.24) | 1.07 (0.54 to 1.87) | 1.17 (0.63 to 2) | 0.2 (0.11 to 0.29) |
| Haiti | 52 (27 to 88) | 329 (128 to 797) | 5.33 (2.05 to 12.56) | 0.81 (0.42 to 1.38) | 2.56 (0.99 to 6.2) | 5.45 (4.17 to 6.75) | 1.1 (0.58 to 1.87) | 2.84 (1.14 to 6.86) | 4.57 (3.45 to 5.71) |
| Honduras | 43 (22 to 73) | 110 (58 to 200) | 1.59 (1.16 to 2.24) | 0.9 (0.47 to 1.55) | 1.09 (0.57 to 1.98) | 0.56 (0.26 to 0.86) | 1.32 (0.69 to 2.23) | 1.3 (0.69 to 2.33) | -0.09 (-0.34 to 0.16) |
| Hungary | 384 (199 to 664) | 284 (150 to 504) | -0.26 (-0.33 to -0.2) | 3.69 (1.91 to 6.39) | 2.96 (1.56 to 5.25) | -1.04 (-1.16 to -0.92) | 2.97 (1.53 to 5.13) | 1.86 (0.98 to 3.35) | -1.84 (-1.96 to -1.72) |
| Iceland | 4 (2 to 7) | 6 (3 to 10) | 0.43 (0.28 to 0.58) | 1.65 (0.85 to 2.84) | 1.71 (0.9 to 2.97) | 0.08 (0.05 to 0.11) | 1.53 (0.79 to 2.63) | 1.23 (0.65 to 2.15) | -0.72 (-0.76 to -0.68) |
| India | 7539 (3929 to 13072) | 15230 (7897 to 26310) | 1.02 (0.92 to 1.14) | 0.88 (0.46 to 1.53) | 1.08 (0.56 to 1.86) | 0.5 (0.44 to 0.55) | 1.29 (0.68 to 2.22) | 1.21 (0.64 to 2.09) | -0.33 (-0.39 to -0.26) |
| Indonesia | 1586 (802 to 2897) | 2245 (1167 to 4031) | 0.42 (0.22 to 0.69) | 0.86 (0.43 to 1.57) | 0.8 (0.42 to 1.45) | -0.26 (-0.38 to -0.14) | 1.11 (0.57 to 1.95) | 0.84 (0.44 to 1.5) | -0.94 (-1.05 to -0.82) |
| Iran (Islamic Republic of) | 1129 (560 to 2111) | 1441 (763 to 2532) | 0.28 (0.11 to 0.46) | 1.98 (0.98 to 3.7) | 1.69 (0.89 to 2.97) | -0.55 (-0.65 to -0.44) | 2.55 (1.33 to 4.68) | 1.6 (0.85 to 2.81) | -1.6 (-1.72 to -1.48) |
| Iraq | 934 (348 to 2209) | 1853 (772 to 4137) | 0.98 (0.79 to 1.29) | 5.07 (1.89 to 11.99) | 4.5 (1.87 to 10.04) | 0.06 (-0.2 to 0.31) | 5.76 (2.25 to 12.99) | 5.28 (2.21 to 11.73) | -0.01 (-0.12 to 0.11) |
| Ireland | 52 (27 to 88) | 80 (42 to 137) | 0.53 (0.38 to 0.69) | 1.45 (0.74 to 2.45) | 1.61 (0.84 to 2.77) | 0.23 (0.12 to 0.34) | 1.38 (0.71 to 2.35) | 1.19 (0.61 to 2.07) | -0.49 (-0.64 to -0.34) |
| Israel | 63 (32 to 107) | 133 (71 to 221) | 1.12 (0.85 to 1.57) | 1.27 (0.65 to 2.16) | 1.39 (0.74 to 2.3) | 0.42 (0.29 to 0.55) | 1.33 (0.68 to 2.27) | 1.24 (0.66 to 2.07) | -0.11 (-0.24 to 0.03) |
| Italy | 1374 (720 to 2341) | 1321 (695 to 2192) | -0.04 (-0.08 to 0.01) | 2.42 (1.27 to 4.12) | 2.21 (1.16 to 3.66) | -0.57 (-0.69 to -0.46) | 1.82 (0.94 to 3.1) | 1.24 (0.64 to 2.09) | -1.45 (-1.55 to -1.35) |
| Jamaica | 16 (9 to 29) | 25 (13 to 42) | 0.51 (0.31 to 0.76) | 0.69 (0.37 to 1.21) | 0.88 (0.47 to 1.5) | 0.58 (0.46 to 0.7) | 0.81 (0.42 to 1.4) | 0.8 (0.43 to 1.37) | -0.25 (-0.39 to -0.12) |
| Japan | 2108 (1086 to 3698) | 2460 (1278 to 4181) | 0.17 (0.1 to 0.24) | 1.68 (0.86 to 2.94) | 1.93 (1 to 3.27) | 0.39 (0.25 to 0.54) | 1.35 (0.7 to 2.39) | 0.97 (0.51 to 1.67) | -1.12 (-1.24 to -1.01) |
| Jordan | 24 (13 to 43) | 81 (42 to 147) | 2.38 (1.87 to 3.07) | 0.64 (0.34 to 1.14) | 0.66 (0.34 to 1.19) | -0.06 (-0.12 to 0.01) | 1.01 (0.54 to 1.8) | 0.77 (0.39 to 1.39) | -0.97 (-1.02 to -0.91) |
| Kazakhstan | 269 (138 to 477) | 286 (149 to 500) | 0.07 (-0.04 to 0.18) | 1.64 (0.84 to 2.91) | 1.51 (0.78 to 2.64) | -0.29 (-0.5 to -0.08) | 1.81 (0.93 to 3.2) | 1.49 (0.77 to 2.61) | -0.64 (-0.81 to -0.48) |
| Kenya | 94 (50 to 166) | 245 (130 to 424) | 1.6 (1.39 to 1.98) | 0.41 (0.21 to 0.72) | 0.49 (0.26 to 0.85) | 0.58 (0.47 to 0.69) | 0.69 (0.37 to 1.19) | 0.71 (0.37 to 1.22) | 0.04 (-0.1 to 0.17) |
| Kiribati | 0 (0 to 1) | 1 (0 to 1) | 0.57 (0.3 to 0.91) | 0.53 (0.27 to 0.93) | 0.51 (0.26 to 0.88) | -0.17 (-0.28 to -0.07) | 0.73 (0.39 to 1.27) | 0.62 (0.33 to 1.09) | -0.49 (-0.59 to -0.39) |
| Kuwait | 18 (9 to 32) | 62 (33 to 109) | 2.44 (1.68 to 3.78) | 1.05 (0.53 to 1.85) | 1.34 (0.71 to 2.35) | -0.1 (-0.4 to 0.21) | 1.32 (0.68 to 2.33) | 1.19 (0.64 to 2.06) | -0.92 (-1.15 to -0.7) |
| Kyrgyzstan | 58 (30 to 101) | 65 (33 to 114) | 0.12 (-0.02 to 0.27) | 1.31 (0.68 to 2.26) | 0.95 (0.48 to 1.67) | -1.09 (-1.15 to -1.04) | 1.63 (0.84 to 2.83) | 1.08 (0.54 to 1.88) | -1.42 (-1.47 to -1.37) |
| Lao People's Democratic Republic | 30 (15 to 52) | 46 (24 to 80) | 0.56 (0.33 to 0.84) | 0.71 (0.37 to 1.25) | 0.62 (0.33 to 1.08) | -0.48 (-0.57 to -0.39) | 1.01 (0.54 to 1.76) | 0.76 (0.4 to 1.32) | -1.04 (-1.09 to -1) |
| Latvia | 104 (53 to 181) | 52 (26 to 92) | -0.5 (-0.56 to -0.45) | 3.9 (2.01 to 6.82) | 2.76 (1.42 to 4.9) | -1.68 (-1.89 to -1.47) | 3.24 (1.67 to 5.67) | 1.78 (0.9 to 3.17) | -2.5 (-2.72 to -2.29) |
| Lebanon | 151 (48 to 429) | 163 (59 to 416) | 0.08 (-0.04 to 0.3) | 5.06 (1.62 to 14.33) | 2.94 (1.06 to 7.51) | -1.79 (-1.88 to -1.69) | 5.24 (1.74 to 14.65) | 2.82 (1.01 to 7.27) | -2.01 (-2.06 to -1.96) |
| Lesotho | 9 (4 to 15) | 16 (8 to 28) | 0.84 (0.59 to 1.1) | 0.57 (0.29 to 1) | 0.85 (0.45 to 1.47) | 1.36 (1.14 to 1.58) | 0.8 (0.41 to 1.4) | 1.09 (0.58 to 1.87) | 1.13 (0.92 to 1.34) |
| Liberia | 20 (10 to 39) | 81 (31 to 183) | 3.04 (1.23 to 5.56) | 0.81 (0.41 to 1.58) | 1.48 (0.58 to 3.36) | 0.93 (0.01 to 1.85) | 0.97 (0.5 to 1.77) | 1.97 (0.8 to 4.4) | 1.5 (0.72 to 2.29) |
| Libya | 45 (23 to 80) | 152 (78 to 277) | 2.4 (1.65 to 3.41) | 1.06 (0.55 to 1.9) | 2.22 (1.13 to 4.03) | 2.74 (2.34 to 3.13) | 1.46 (0.76 to 2.56) | 2.14 (1.11 to 3.83) | 1.53 (1.22 to 1.85) |
| Lithuania | 121 (62 to 211) | 84 (43 to 146) | -0.31 (-0.37 to -0.23) | 3.29 (1.69 to 5.74) | 3.06 (1.58 to 5.36) | -0.42 (-0.61 to -0.22) | 2.88 (1.48 to 5) | 1.91 (0.98 to 3.33) | -1.57 (-1.78 to -1.36) |
| Luxembourg | 11 (6 to 20) | 14 (7 to 24) | 0.22 (0.1 to 0.34) | 2.96 (1.54 to 5.17) | 2.13 (1.12 to 3.79) | -1.07 (-1.22 to -0.92) | 2.28 (1.18 to 3.95) | 1.46 (0.76 to 2.61) | -1.47 (-1.59 to -1.35) |
| Madagascar | 49 (25 to 89) | 104 (52 to 180) | 1.11 (0.73 to 1.55) | 0.41 (0.21 to 0.75) | 0.36 (0.18 to 0.63) | -0.44 (-0.52 to -0.36) | 0.62 (0.31 to 1.09) | 0.54 (0.28 to 0.92) | -0.49 (-0.53 to -0.44) |
| Malawi | 39 (20 to 67) | 68 (35 to 119) | 0.77 (0.48 to 1.13) | 0.39 (0.2 to 0.68) | 0.35 (0.18 to 0.61) | -0.54 (-0.6 to -0.49) | 0.61 (0.32 to 1.06) | 0.56 (0.29 to 0.96) | -0.38 (-0.46 to -0.31) |
| Malaysia | 100 (51 to 173) | 225 (113 to 398) | 1.26 (0.94 to 1.62) | 0.56 (0.29 to 0.98) | 0.71 (0.36 to 1.25) | 0.7 (0.63 to 0.77) | 0.78 (0.41 to 1.34) | 0.73 (0.37 to 1.29) | -0.26 (-0.27 to -0.24) |
| Maldives | 1 (1 to 2) | 4 (2 to 7) | 2.27 (1.7 to 3.05) | 0.52 (0.27 to 0.91) | 0.73 (0.38 to 1.31) | 1.43 (1.21 to 1.65) | 0.83 (0.44 to 1.44) | 0.77 (0.4 to 1.36) | -0.05 (-0.2 to 0.1) |
| Mali | 45 (23 to 79) | 191 (90 to 377) | 3.2 (1.85 to 5.73) | 0.52 (0.27 to 0.91) | 0.79 (0.37 to 1.56) | 1.24 (0.5 to 1.98) | 0.79 (0.41 to 1.36) | 1.25 (0.6 to 2.4) | 1.53 (1.07 to 1.99) |
| Malta | 6 (3 to 11) | 9 (5 to 17) | 0.47 (0.33 to 0.64) | 1.73 (0.91 to 3) | 2.13 (1.13 to 3.74) | 0.82 (0.74 to 0.91) | 1.57 (0.82 to 2.71) | 1.29 (0.68 to 2.25) | -0.51 (-0.61 to -0.42) |
| Marshall Islands | 0 (0 to 0) | 0 (0 to 1) | 0.66 (0.44 to 0.93) | 0.5 (0.26 to 0.91) | 0.67 (0.35 to 1.16) | 1 (0.94 to 1.05) | 0.87 (0.46 to 1.57) | 0.81 (0.43 to 1.41) | -0.24 (-0.31 to -0.18) |
| Mauritania | 11 (6 to 19) | 20 (10 to 34) | 0.74 (0.47 to 1.05) | 0.55 (0.27 to 0.93) | 0.44 (0.23 to 0.77) | -0.71 (-0.75 to -0.66) | 0.82 (0.41 to 1.38) | 0.66 (0.35 to 1.13) | -0.73 (-0.76 to -0.69) |
| Mauritius | 6 (3 to 11) | 10 (5 to 18) | 0.67 (0.41 to 0.96) | 0.56 (0.29 to 0.99) | 0.8 (0.41 to 1.42) | 1.38 (1.31 to 1.46) | 0.65 (0.34 to 1.16) | 0.62 (0.32 to 1.1) | 0 (-0.06 to 0.07) |
| Mexico | 1206 (631 to 2108) | 1670 (885 to 2874) | 0.39 (0.31 to 0.46) | 1.41 (0.74 to 2.47) | 1.29 (0.68 to 2.22) | 0.62 (0.26 to 0.99) | 1.98 (1.04 to 3.43) | 1.27 (0.67 to 2.18) | -0.61 (-0.95 to -0.27) |
| Micronesia (Federated States of) | 1 (0 to 1) | 1 (0 to 1) | 0.38 (0.19 to 0.62) | 0.6 (0.31 to 1.05) | 0.83 (0.44 to 1.44) | 1.22 (1.11 to 1.34) | 0.93 (0.48 to 1.64) | 0.96 (0.51 to 1.68) | 0.2 (0.11 to 0.29) |
| Monaco | 1 (0 to 1) | 1 (0 to 1) | 0.25 (0.14 to 0.38) | 2.04 (1.09 to 3.56) | 2.05 (1.06 to 3.54) | 0.03 (-0.06 to 0.12) | 1.21 (0.64 to 2.15) | 1.13 (0.59 to 1.94) | -0.15 (-0.25 to -0.05) |
| Mongolia | 24 (12 to 42) | 50 (26 to 86) | 1.06 (0.82 to 1.32) | 1.12 (0.58 to 1.95) | 1.48 (0.78 to 2.58) | 1.14 (1.02 to 1.27) | 1.59 (0.83 to 2.78) | 1.64 (0.86 to 2.83) | 0.15 (0.08 to 0.22) |
| Montenegro | 14 (7 to 24) | 15 (8 to 26) | 0.06 (-0.04 to 0.17) | 2.2 (1.13 to 3.81) | 2.37 (1.21 to 4.21) | 0.23 (0.12 to 0.34) | 2.16 (1.11 to 3.74) | 1.81 (0.92 to 3.22) | -0.6 (-0.69 to -0.52) |
| Morocco | 263 (138 to 461) | 398 (208 to 697) | 0.52 (0.32 to 0.75) | 1.04 (0.54 to 1.82) | 1.07 (0.56 to 1.88) | 0 (-0.07 to 0.06) | 1.36 (0.71 to 2.36) | 1.08 (0.57 to 1.89) | -0.84 (-0.94 to -0.74) |
| Mozambique | 335 (118 to 837) | 314 (137 to 700) | -0.06 (-0.22 to 0.33) | 2.51 (0.89 to 6.26) | 1.01 (0.44 to 2.25) | -3.09 (-3.18 to -3) | 2.77 (1.02 to 6.58) | 1.88 (0.79 to 4.2) | -1.29 (-1.37 to -1.2) |
| Myanmar | 476 (214 to 1038) | 881 (438 to 1688) | 0.85 (0.3 to 1.82) | 1.18 (0.53 to 2.57) | 1.56 (0.78 to 2.99) | 1.12 (0.82 to 1.42) | 1.38 (0.65 to 2.82) | 1.61 (0.8 to 3.11) | 0.74 (0.45 to 1.03) |
| Namibia | 20 (9 to 41) | 23 (12 to 41) | 0.14 (-0.12 to 0.58) | 1.4 (0.61 to 2.94) | 0.93 (0.48 to 1.68) | -1.38 (-1.41 to -1.34) | 1.65 (0.77 to 3.18) | 1.22 (0.63 to 2.24) | -1.04 (-1.15 to -0.92) |
| Nauru | 0 (0 to 0) | 0 (0 to 0) | 0.19 (0.04 to 0.36) | 0.69 (0.35 to 1.24) | 0.76 (0.39 to 1.31) | 0.2 (0.03 to 0.37) | 1.02 (0.53 to 1.8) | 1.04 (0.55 to 1.8) | -0.03 (-0.16 to 0.11) |
| Nepal | 136 (71 to 235) | 402 (209 to 737) | 1.96 (1.38 to 2.85) | 0.7 (0.36 to 1.21) | 1.29 (0.67 to 2.37) | 2.47 (2.15 to 2.8) | 1.04 (0.55 to 1.8) | 1.48 (0.77 to 2.69) | 1.41 (1.22 to 1.61) |
| Netherlands | 215 (112 to 368) | 322 (172 to 561) | 0.5 (0.36 to 0.69) | 1.44 (0.75 to 2.47) | 1.87 (1 to 3.26) | 1.05 (0.72 to 1.38) | 1.17 (0.61 to 2.03) | 1.11 (0.58 to 1.93) | -0.01 (-0.27 to 0.25) |
| New Zealand | 94 (48 to 163) | 144 (74 to 244) | 0.54 (0.42 to 0.66) | 2.75 (1.4 to 4.78) | 2.79 (1.44 to 4.72) | 0.18 (0.13 to 0.24) | 2.54 (1.3 to 4.41) | 2.07 (1.08 to 3.51) | -0.57 (-0.61 to -0.53) |
| Nicaragua | 180 (58 to 425) | 169 (70 to 362) | -0.07 (-0.18 to 0.24) | 4.64 (1.48 to 10.93) | 2.53 (1.05 to 5.42) | -1.9 (-1.95 to -1.84) | 4.78 (1.71 to 10.82) | 2.94 (1.21 to 6.33) | -1.58 (-1.67 to -1.49) |
| Niger | 38 (20 to 66) | 118 (61 to 209) | 2.1 (1.5 to 2.9) | 0.47 (0.25 to 0.83) | 0.47 (0.25 to 0.84) | -0.29 (-0.42 to -0.17) | 0.78 (0.42 to 1.33) | 0.79 (0.41 to 1.36) | -0.07 (-0.14 to 0) |
| Nigeria | 452 (236 to 781) | 1130 (611 to 2007) | 1.5 (1.19 to 2.07) | 0.5 (0.26 to 0.87) | 0.49 (0.26 to 0.87) | -0.12 (-0.16 to -0.08) | 0.73 (0.38 to 1.25) | 0.74 (0.4 to 1.27) | 0.01 (-0.04 to 0.06) |
| Niue | 0 (0 to 0) | 0 (0 to 0) | -0.11 (-0.23 to 0.02) | 0.74 (0.39 to 1.28) | 0.91 (0.47 to 1.54) | 0.64 (0.42 to 0.86) | 0.8 (0.42 to 1.39) | 0.79 (0.41 to 1.35) | -0.07 (-0.2 to 0.07) |
| North Macedonia | 32 (16 to 57) | 44 (22 to 80) | 0.36 (0.22 to 0.53) | 1.61 (0.83 to 2.84) | 2.01 (1.03 to 3.66) | 0.77 (0.62 to 0.92) | 1.61 (0.83 to 2.84) | 1.57 (0.81 to 2.87) | -0.05 (-0.21 to 0.1) |
| Northern Mariana Islands | 0 (0 to 1) | 1 (0 to 1) | 0.51 (0.29 to 0.78) | 0.77 (0.39 to 1.42) | 1.07 (0.56 to 1.88) | 1.28 (1.15 to 1.42) | 1.04 (0.54 to 1.81) | 1 (0.53 to 1.78) | -0.17 (-0.21 to -0.14) |
| Norway | 95 (50 to 162) | 100 (53 to 171) | 0.05 (-0.01 to 0.12) | 2.24 (1.18 to 3.81) | 1.85 (0.98 to 3.16) | -0.67 (-0.75 to -0.58) | 1.66 (0.87 to 2.86) | 1.19 (0.63 to 2.06) | -1.05 (-1.15 to -0.96) |
| Oman | 27 (14 to 47) | 56 (29 to 99) | 1.08 (0.83 to 1.42) | 1.36 (0.71 to 2.35) | 1.19 (0.61 to 2.11) | -0.47 (-0.54 to -0.4) | 2.09 (1.12 to 3.61) | 1.42 (0.73 to 2.46) | -1.42 (-1.57 to -1.27) |
| Pakistan | 497 (259 to 871) | 1677 (853 to 3113) | 2.37 (1.57 to 3.89) | 0.45 (0.23 to 0.78) | 0.71 (0.36 to 1.32) | 1.96 (1.73 to 2.18) | 0.65 (0.34 to 1.12) | 0.85 (0.44 to 1.56) | 1.15 (0.99 to 1.31) |
| Palau | 0 (0 to 0) | 0 (0 to 0) | 0.82 (0.62 to 1.1) | 0.98 (0.5 to 1.73) | 1.49 (0.78 to 2.63) | 1.33 (1.28 to 1.37) | 1.23 (0.64 to 2.17) | 1.23 (0.65 to 2.17) | 0.02 (0 to 0.04) |
| Palestine | 70 (30 to 159) | 183 (75 to 406) | 1.6 (0.99 to 2.1) | 3.44 (1.46 to 7.75) | 3.56 (1.46 to 7.9) | 1.02 (0.59 to 1.46) | 3.91 (1.7 to 8.64) | 4.46 (1.84 to 9.73) | 1.05 (0.74 to 1.37) |
| Panama | 26 (13 to 47) | 45 (23 to 81) | 0.71 (0.41 to 0.97) | 1.1 (0.54 to 1.98) | 1.04 (0.54 to 1.88) | -0.17 (-0.22 to -0.12) | 1.28 (0.66 to 2.28) | 1.01 (0.52 to 1.83) | -0.78 (-0.83 to -0.72) |
| Papua New Guinea | 23 (12 to 41) | 85 (44 to 153) | 2.62 (2.03 to 3.41) | 0.57 (0.29 to 0.99) | 0.81 (0.42 to 1.46) | 1 (0.77 to 1.22) | 0.89 (0.47 to 1.55) | 1.15 (0.61 to 2.04) | 0.72 (0.55 to 0.88) |
| Paraguay | 34 (17 to 60) | 73 (39 to 125) | 1.14 (0.84 to 1.5) | 0.84 (0.43 to 1.48) | 1.02 (0.55 to 1.75) | 0.63 (0.56 to 0.71) | 1.14 (0.59 to 2) | 1.1 (0.59 to 1.88) | -0.13 (-0.23 to -0.04) |
| Peru | 255 (124 to 477) | 404 (207 to 726) | 0.58 (0.35 to 0.88) | 1.18 (0.57 to 2.21) | 1.11 (0.57 to 2) | -0.29 (-0.34 to -0.24) | 1.35 (0.68 to 2.45) | 1.12 (0.57 to 2.01) | -0.76 (-0.83 to -0.68) |
| Philippines | 643 (316 to 1226) | 1066 (546 to 1946) | 0.66 (0.41 to 0.91) | 1.02 (0.5 to 1.95) | 0.94 (0.48 to 1.72) | -0.32 (-0.41 to -0.23) | 1.29 (0.66 to 2.38) | 1.05 (0.54 to 1.92) | -0.72 (-0.76 to -0.68) |
| Poland | 1025 (533 to 1777) | 1036 (539 to 1809) | 0.01 (-0.04 to 0.06) | 2.68 (1.4 to 4.66) | 2.71 (1.41 to 4.73) | -0.02 (-0.1 to 0.05) | 2.47 (1.28 to 4.29) | 1.82 (0.93 to 3.19) | -1.1 (-1.19 to -1.02) |
| Portugal | 223 (119 to 378) | 197 (105 to 336) | -0.12 (-0.21 to -0.02) | 2.2 (1.18 to 3.73) | 1.86 (0.99 to 3.17) | -0.73 (-0.83 to -0.64) | 1.86 (0.99 to 3.18) | 1.05 (0.55 to 1.82) | -2.04 (-2.12 to -1.96) |
| Puerto Rico | 39 (20 to 68) | 56 (30 to 101) | 0.46 (0.26 to 0.69) | 1.07 (0.56 to 1.87) | 1.71 (0.91 to 3.06) | 1.48 (1.44 to 1.52) | 1.07 (0.56 to 1.88) | 1.08 (0.57 to 1.95) | -0.02 (-0.06 to 0.02) |
| Qatar | 5 (3 to 9) | 34 (17 to 62) | 5.49 (4.38 to 7.12) | 1.18 (0.62 to 2.12) | 1.15 (0.58 to 2.07) | -0.11 (-0.33 to 0.11) | 1.47 (0.76 to 2.62) | 1.16 (0.59 to 2.09) | -0.78 (-0.94 to -0.63) |
| Republic of Korea | 842 (434 to 1517) | 1033 (546 to 1803) | 0.23 (0.09 to 0.38) | 1.9 (0.98 to 3.43) | 2 (1.06 to 3.5) | -0.11 (-0.21 to -0.01) | 2.28 (1.18 to 4.12) | 1.27 (0.67 to 2.24) | -2.11 (-2.19 to -2.04) |
| Republic of Moldova | 107 (57 to 186) | 73 (37 to 128) | -0.32 (-0.38 to -0.25) | 2.41 (1.29 to 4.18) | 2.02 (1.03 to 3.56) | -0.7 (-0.77 to -0.64) | 2.39 (1.27 to 4.12) | 1.46 (0.75 to 2.57) | -1.75 (-1.82 to -1.68) |
| Romania | 703 (365 to 1231) | 535 (265 to 940) | -0.24 (-0.31 to -0.16) | 3.01 (1.56 to 5.27) | 2.82 (1.4 to 4.97) | -0.31 (-0.36 to -0.26) | 2.71 (1.41 to 4.77) | 1.93 (0.97 to 3.41) | -1.26 (-1.32 to -1.2) |
| Russian Federation | 4699 (2443 to 8161) | 4330 (2236 to 7470) | -0.08 (-0.12 to -0.03) | 3.11 (1.62 to 5.41) | 2.99 (1.54 to 5.16) | -0.46 (-0.86 to -0.05) | 2.76 (1.44 to 4.79) | 2.19 (1.12 to 3.76) | -1.06 (-1.44 to -0.69) |
| Rwanda | 43 (22 to 77) | 564 (172 to 1419) | 11.97 (3.96 to 28.75) | 0.6 (0.31 to 1.07) | 4.25 (1.29 to 10.69) | 2.9 (0.05 to 5.84) | 0.89 (0.46 to 1.57) | 5.61 (1.74 to 14.18) | 3.31 (0.89 to 5.8) |
| Saint Kitts and Nevis | 0 (0 to 1) | 1 (0 to 1) | 0.84 (0.62 to 1.12) | 0.87 (0.46 to 1.54) | 1.14 (0.6 to 1.88) | 1.02 (0.93 to 1.12) | 1 (0.53 to 1.74) | 0.97 (0.51 to 1.61) | 0.05 (-0.07 to 0.16) |
| Saint Lucia | 1 (0 to 2) | 2 (1 to 3) | 0.98 (0.7 to 1.3) | 0.68 (0.35 to 1.18) | 1.03 (0.53 to 1.8) | 1.43 (1.37 to 1.49) | 0.87 (0.46 to 1.51) | 0.83 (0.43 to 1.46) | -0.14 (-0.22 to -0.06) |
| Saint Vincent and the Grenadines | 1 (0 to 1) | 1 (1 to 2) | 0.6 (0.38 to 0.83) | 0.67 (0.34 to 1.18) | 1.02 (0.54 to 1.77) | 1.4 (1.31 to 1.5) | 0.85 (0.44 to 1.5) | 0.89 (0.47 to 1.55) | 0.13 (0.04 to 0.23) |
| Samoa | 1 (0 to 2) | 2 (1 to 3) | 0.82 (0.43 to 1.5) | 0.53 (0.28 to 0.96) | 0.77 (0.4 to 1.44) | 1.59 (1.3 to 1.88) | 0.81 (0.43 to 1.44) | 0.96 (0.5 to 1.78) | 0.86 (0.64 to 1.09) |
| San Marino | 0 (0 to 1) | 1 (0 to 1) | 0.75 (0.59 to 0.95) | 1.64 (0.87 to 2.81) | 2.09 (1.08 to 3.66) | 0.8 (0.71 to 0.89) | 1.28 (0.68 to 2.21) | 1.19 (0.62 to 2.1) | -0.19 (-0.27 to -0.11) |
| Sao Tome and Principe | 1 (0 to 1) | 1 (1 to 2) | 1.07 (0.73 to 1.44) | 0.5 (0.26 to 0.87) | 0.58 (0.3 to 1.01) | 0.35 (0.24 to 0.47) | 0.72 (0.37 to 1.24) | 0.82 (0.43 to 1.41) | 0.33 (0.31 to 0.36) |
| Saudi Arabia | 220 (111 to 387) | 875 (451 to 1582) | 2.98 (2.53 to 3.49) | 1.39 (0.7 to 2.44) | 2.32 (1.19 to 4.2) | 1.79 (1.68 to 1.89) | 2.24 (1.15 to 3.9) | 2.56 (1.34 to 4.49) | 0.6 (0.53 to 0.66) |
| Senegal | 29 (15 to 51) | 67 (35 to 118) | 1.29 (0.91 to 1.85) | 0.38 (0.2 to 0.67) | 0.42 (0.22 to 0.75) | 0.2 (0.06 to 0.34) | 0.6 (0.31 to 1.04) | 0.62 (0.32 to 1.09) | 0.06 (-0.03 to 0.15) |
| Serbia | 211 (109 to 363) | 231 (123 to 399) | 0.09 (-0.06 to 0.35) | 2.19 (1.13 to 3.77) | 2.59 (1.38 to 4.48) | 0.53 (0.34 to 0.72) | 1.98 (1.02 to 3.42) | 1.87 (0.98 to 3.25) | -0.23 (-0.45 to -0.01) |
| Seychelles | 1 (0 to 1) | 1 (0 to 1) | 0.65 (0.41 to 0.92) | 0.71 (0.37 to 1.24) | 0.81 (0.42 to 1.41) | 0.42 (0.38 to 0.45) | 0.83 (0.43 to 1.41) | 0.71 (0.37 to 1.25) | -0.48 (-0.51 to -0.45) |
| Sierra Leone | 19 (10 to 34) | 93 (42 to 194) | 3.89 (1.78 to 7.94) | 0.46 (0.24 to 0.81) | 1.05 (0.47 to 2.18) | 1.57 (0.42 to 2.73) | 0.64 (0.33 to 1.11) | 1.49 (0.68 to 3.06) | 2.02 (1.14 to 2.91) |
| Singapore | 37 (19 to 65) | 83 (42 to 142) | 1.22 (0.97 to 1.49) | 1.22 (0.63 to 2.13) | 1.44 (0.74 to 2.48) | 0.43 (0.38 to 0.48) | 1.34 (0.68 to 2.31) | 1.05 (0.54 to 1.81) | -0.85 (-0.93 to -0.77) |
| Slovakia | 156 (79 to 270) | 168 (86 to 292) | 0.08 (-0.01 to 0.18) | 2.95 (1.5 to 5.11) | 3.09 (1.59 to 5.37) | 0.19 (0.12 to 0.26) | 2.74 (1.39 to 4.75) | 2.15 (1.11 to 3.72) | -0.79 (-0.85 to -0.73) |
| Slovenia | 75 (39 to 129) | 82 (43 to 143) | 0.09 (0 to 0.21) | 3.8 (1.95 to 6.55) | 3.96 (2.06 to 6.92) | 0.46 (0.27 to 0.64) | 3.25 (1.68 to 5.59) | 2.35 (1.23 to 4.09) | -0.75 (-0.95 to -0.55) |
| Solomon Islands | 2 (1 to 4) | 6 (3 to 11) | 1.64 (1.31 to 2.04) | 0.71 (0.37 to 1.29) | 0.93 (0.48 to 1.68) | 0.88 (0.84 to 0.91) | 1.24 (0.65 to 2.2) | 1.39 (0.72 to 2.47) | 0.32 (0.29 to 0.34) |
| Somalia | 74 (27 to 243) | 267 (107 to 578) | 2.62 (0.7 to 6.2) | 0.93 (0.34 to 3.06) | 1.23 (0.5 to 2.67) | 0.31 (0.04 to 0.58) | 1.2 (0.5 to 3.31) | 1.78 (0.74 to 4) | 0.78 (0.65 to 0.92) |
| South Africa | 461 (242 to 822) | 557 (295 to 1008) | 0.21 (0.13 to 0.29) | 1.25 (0.65 to 2.22) | 0.98 (0.52 to 1.77) | -1.03 (-1.18 to -0.89) | 1.62 (0.86 to 2.82) | 1.01 (0.53 to 1.82) | -1.74 (-1.9 to -1.59) |
| South Sudan | 55 (24 to 110) | 130 (53 to 294) | 1.38 (0.93 to 1.83) | 0.93 (0.4 to 1.86) | 1.35 (0.55 to 3.04) | 1.42 (1 to 1.85) | 1.1 (0.51 to 2.1) | 1.79 (0.76 to 4.01) | 1.82 (1.51 to 2.13) |
| Spain | 710 (368 to 1221) | 884 (454 to 1514) | 0.25 (0.12 to 0.37) | 1.83 (0.95 to 3.15) | 1.94 (1 to 3.32) | 0.06 (-0.01 to 0.12) | 1.51 (0.77 to 2.6) | 1.16 (0.6 to 2.04) | -0.88 (-0.97 to -0.8) |
| Sri Lanka | 259 (128 to 505) | 683 (320 to 1432) | 1.64 (1.02 to 2.47) | 1.51 (0.75 to 2.95) | 3.07 (1.44 to 6.43) | 2.02 (1.69 to 2.34) | 1.64 (0.83 to 3.1) | 2.79 (1.29 to 5.9) | 1.62 (1.29 to 1.95) |
| Sudan | 216 (109 to 394) | 548 (266 to 1079) | 1.54 (0.99 to 2.16) | 1.08 (0.54 to 1.97) | 1.26 (0.61 to 2.49) | 0.63 (0.4 to 0.86) | 1.41 (0.72 to 2.5) | 1.66 (0.83 to 3.15) | 0.65 (0.45 to 0.86) |
| Suriname | 4 (2 to 9) | 6 (3 to 12) | 0.56 (0.21 to 0.95) | 1.03 (0.49 to 2.22) | 1.07 (0.55 to 1.99) | 0.18 (0.15 to 0.22) | 1.13 (0.56 to 2.2) | 0.99 (0.5 to 1.82) | -0.38 (-0.43 to -0.32) |
| Sweden | 180 (96 to 300) | 198 (104 to 337) | 0.1 (0 to 0.19) | 2.1 (1.11 to 3.49) | 1.9 (1.01 to 3.25) | -0.31 (-0.43 to -0.19) | 1.46 (0.77 to 2.46) | 1.15 (0.6 to 1.97) | -0.77 (-0.88 to -0.67) |
| Switzerland | 222 (117 to 382) | 208 (109 to 353) | -0.06 (-0.14 to 0.03) | 3.23 (1.7 to 5.56) | 2.33 (1.22 to 3.96) | -1.21 (-1.36 to -1.05) | 2.34 (1.22 to 4.04) | 1.41 (0.74 to 2.44) | -1.79 (-1.88 to -1.7) |
| Syrian Arab Republic | 116 (55 to 238) | 732 (276 to 1632) | 5.34 (2.39 to 10.85) | 0.91 (0.44 to 1.87) | 5.22 (1.97 to 11.63) | 6.47 (4.76 to 8.2) | 1.26 (0.63 to 2.51) | 5.85 (2.17 to 12.88) | 5.35 (3.84 to 6.88) |
| Taiwan (Province of China) | 215 (113 to 376) | 247 (133 to 437) | 0.15 (0 to 0.32) | 1.06 (0.56 to 1.84) | 1.05 (0.56 to 1.85) | -0.43 (-0.56 to -0.3) | 1.19 (0.63 to 2.08) | 0.68 (0.37 to 1.21) | -2.19 (-2.33 to -2.05) |
| Tajikistan | 51 (27 to 91) | 145 (72 to 280) | 1.85 (0.9 to 3.8) | 0.95 (0.5 to 1.7) | 1.43 (0.71 to 2.76) | -0.31 (-1.1 to 0.49) | 1.34 (0.71 to 2.41) | 1.72 (0.85 to 3.3) | -0.38 (-0.98 to 0.22) |
| Thailand | 590 (312 to 1021) | 962 (510 to 1693) | 0.63 (0.43 to 0.86) | 1.04 (0.55 to 1.8) | 1.44 (0.77 to 2.54) | 0.78 (0.69 to 0.88) | 1.24 (0.65 to 2.16) | 1.03 (0.54 to 1.83) | -0.87 (-0.95 to -0.78) |
| Timor-Leste | 31 (10 to 79) | 42 (15 to 103) | 0.37 (0.25 to 0.51) | 3.96 (1.32 to 10.12) | 3.03 (1.04 to 7.39) | -1.17 (-1.66 to -0.68) | 3.97 (1.44 to 9.62) | 4.36 (1.47 to 10.77) | 0.16 (-0.31 to 0.63) |
| Togo | 16 (8 to 28) | 41 (21 to 73) | 1.57 (1.17 to 2.06) | 0.44 (0.22 to 0.75) | 0.49 (0.25 to 0.87) | 0.33 (0.26 to 0.41) | 0.73 (0.37 to 1.29) | 0.71 (0.37 to 1.25) | -0.1 (-0.16 to -0.05) |
| Tokelau | 0 (0 to 0) | 0 (0 to 0) | -0.04 (-0.16 to 0.11) | 0.63 (0.33 to 1.09) | 0.71 (0.37 to 1.24) | 0.43 (0.27 to 0.58) | 0.76 (0.39 to 1.3) | 0.69 (0.36 to 1.2) | -0.35 (-0.42 to -0.28) |
| Tonga | 0 (0 to 1) | 0 (0 to 1) | 0.1 (-0.06 to 0.29) | 0.45 (0.23 to 0.79) | 0.47 (0.24 to 0.82) | 0.1 (0.02 to 0.19) | 0.66 (0.34 to 1.14) | 0.56 (0.29 to 0.98) | -0.49 (-0.56 to -0.41) |
| Trinidad and Tobago | 9 (5 to 16) | 16 (9 to 28) | 0.8 (0.52 to 1.12) | 0.76 (0.39 to 1.33) | 1.18 (0.63 to 2.02) | 1.58 (1.41 to 1.76) | 0.89 (0.46 to 1.55) | 0.99 (0.53 to 1.7) | 0.46 (0.32 to 0.59) |
| Tunisia | 80 (42 to 146) | 135 (73 to 239) | 0.68 (0.49 to 0.95) | 0.96 (0.51 to 1.75) | 1.14 (0.61 to 2.02) | 0.58 (0.54 to 0.61) | 1.23 (0.65 to 2.2) | 1.03 (0.55 to 1.81) | -0.6 (-0.64 to -0.57) |
| Turkmenistan | 35 (18 to 64) | 49 (25 to 84) | 0.38 (0.2 to 0.62) | 0.95 (0.49 to 1.72) | 0.94 (0.49 to 1.62) | -0.17 (-0.23 to -0.1) | 1.26 (0.65 to 2.25) | 1 (0.52 to 1.71) | -0.92 (-0.97 to -0.86) |
| Tuvalu | 0 (0 to 0) | 0 (0 to 0) | 0.3 (0.13 to 0.52) | 0.76 (0.4 to 1.33) | 0.76 (0.4 to 1.32) | -0.53 (-0.76 to -0.31) | 0.93 (0.49 to 1.61) | 0.85 (0.44 to 1.45) | -0.79 (-0.99 to -0.59) |
| Uganda | 331 (118 to 812) | 357 (158 to 719) | 0.08 (-0.13 to 0.57) | 1.91 (0.68 to 4.69) | 0.83 (0.37 to 1.66) | -2.71 (-2.76 to -2.66) | 2.2 (0.85 to 5.03) | 1.57 (0.67 to 3.31) | -1.11 (-1.23 to -0.98) |
| Ukraine | 1717 (901 to 3019) | 1367 (718 to 2373) | -0.2 (-0.27 to -0.13) | 3.26 (1.71 to 5.73) | 3.17 (1.67 to 5.51) | -0.62 (-0.83 to -0.41) | 2.72 (1.42 to 4.8) | 2.25 (1.18 to 3.92) | -1.13 (-1.33 to -0.93) |
| United Arab Emirates | 21 (10 to 36) | 156 (77 to 278) | 6.43 (5.1 to 8.04) | 1.12 (0.55 to 1.92) | 1.62 (0.8 to 2.88) | 0.97 (0.74 to 1.2) | 1.5 (0.77 to 2.6) | 1.36 (0.7 to 2.46) | -0.38 (-0.4 to -0.36) |
| United Kingdom | 1018 (529 to 1731) | 1131 (593 to 1903) | 0.11 (0.07 to 0.16) | 1.78 (0.92 to 3.02) | 1.67 (0.87 to 2.8) | -0.17 (-0.26 to -0.08) | 1.34 (0.7 to 2.3) | 1.08 (0.56 to 1.82) | -0.67 (-0.76 to -0.57) |
| United Republic of Tanzania | 112 (57 to 198) | 245 (130 to 426) | 1.19 (0.85 to 1.62) | 0.43 (0.22 to 0.76) | 0.42 (0.22 to 0.73) | -0.12 (-0.16 to -0.09) | 0.67 (0.35 to 1.2) | 0.63 (0.34 to 1.09) | -0.23 (-0.27 to -0.2) |
| United States of America | 4291 (2203 to 7433) | 6471 (3451 to 11065) | 0.51 (0.39 to 0.62) | 1.69 (0.87 to 2.93) | 1.95 (1.04 to 3.33) | 0.49 (0.37 to 0.6) | 1.46 (0.75 to 2.54) | 1.28 (0.68 to 2.2) | -0.39 (-0.47 to -0.3) |
| United States Virgin Islands | 1 (1 to 2) | 1 (1 to 2) | 0.13 (-0.01 to 0.32) | 0.94 (0.48 to 1.62) | 1.31 (0.69 to 2.29) | 0.79 (0.67 to 0.92) | 1.01 (0.52 to 1.76) | 0.85 (0.44 to 1.49) | -0.82 (-1 to -0.64) |
| Uruguay | 53 (27 to 91) | 58 (29 to 99) | 0.09 (-0.02 to 0.19) | 1.69 (0.86 to 2.91) | 1.7 (0.86 to 2.9) | -0.1 (-0.15 to -0.06) | 1.54 (0.78 to 2.64) | 1.3 (0.65 to 2.23) | -0.66 (-0.7 to -0.61) |
| Uzbekistan | 197 (103 to 346) | 337 (177 to 607) | 0.71 (0.48 to 0.96) | 0.94 (0.49 to 1.65) | 0.98 (0.52 to 1.77) | 0.18 (0.06 to 0.29) | 1.26 (0.66 to 2.2) | 1.05 (0.55 to 1.87) | -0.65 (-0.76 to -0.54) |
| Vanuatu | 1 (0 to 1) | 2 (1 to 3) | 1.3 (0.97 to 1.76) | 0.53 (0.27 to 0.96) | 0.59 (0.31 to 1.06) | 0.33 (0.21 to 0.44) | 0.8 (0.42 to 1.45) | 0.79 (0.42 to 1.4) | -0.09 (-0.19 to 0.01) |
| Venezuela (Bolivarian Republic of) | 218 (116 to 383) | 424 (227 to 733) | 0.94 (0.68 to 1.23) | 1.16 (0.61 to 2.03) | 1.59 (0.85 to 2.75) | 0.96 (0.84 to 1.08) | 1.51 (0.8 to 2.6) | 1.46 (0.78 to 2.51) | -0.09 (-0.22 to 0.03) |
| Viet Nam | 436 (228 to 773) | 1005 (537 to 1750) | 1.3 (1.02 to 1.62) | 0.64 (0.33 to 1.13) | 1 (0.54 to 1.75) | 1.74 (1.63 to 1.85) | 0.9 (0.47 to 1.58) | 1 (0.54 to 1.73) | 0.53 (0.44 to 0.62) |
| Yemen | 144 (71 to 268) | 690 (312 to 1443) | 3.8 (2.33 to 5.48) | 1.05 (0.52 to 1.96) | 2.05 (0.93 to 4.29) | 1.48 (0.81 to 2.17) | 1.59 (0.8 to 2.87) | 2.38 (1.14 to 4.85) | 0.78 (0.36 to 1.2) |
| Zambia | 33 (17 to 59) | 80 (42 to 139) | 1.39 (0.98 to 1.87) | 0.42 (0.22 to 0.74) | 0.41 (0.22 to 0.71) | -0.19 (-0.24 to -0.15) | 0.69 (0.36 to 1.22) | 0.68 (0.36 to 1.19) | -0.12 (-0.21 to -0.03) |
| Zimbabwe | 47 (25 to 83) | 76 (40 to 132) | 0.6 (0.34 to 0.93) | 0.46 (0.24 to 0.8) | 0.48 (0.25 to 0.84) | 0.12 (0.06 to 0.18) | 0.76 (0.4 to 1.33) | 0.72 (0.38 to 1.23) | -0.23 (-0.3 to -0.17) |

YLDs, years lived with disability; CR, crude rate; ASR, age-standardized rate; EAPC, estimated annual percentage change; UI, uncertainty interval; CI, confidence interval.
